# A Multi-Fish Tracking and Behavior Modeling Framework for High-Density Cage Aquaculture

**DOI:** 10.3390/s26010256

**Published:** 2025-12-31

**Authors:** Xinyao Xiao, Tao Liu, Shuangyan He, Peiliang Li, Yanzhen Gu, Pixue Li, Jiang Dong

**Affiliations:** 1 State Key Laboratory of Ocean Sensing & Ocean College, Zhejiang University, Zhoushan 316021, China; 2 Hainan Institute, Zhejiang University, Sanya 572025, China; 3 Hainan Observation and Research Station of Ecological Environment and Fishery Resource in Yazhou Bay, Sanya 572025, China; 4 State Key Laboratory of Ocean Sensing, ZJU-Hangzhou Global Scientific and Technological Innovation Center, Zhejiang University, Hangzhou 311215, China; 5 Shanghai Marine Monitoring and Forecasting Center, Shanghai 200062, China; 6 Northern Navigation Service Center of Maritime Safety Administration, Tianjin 300220, China

**Keywords:** behavioral modeling, extended kalman filter, multi-object tracking, cage aquaculture

## Abstract

Multi-fish tracking and behavior analysis in deep-sea cages face two critical challenges: first, the homogeneity of fish appearance and low image quality render appearance-based association unreliable; second, standard linear motion models fail to capture the complex, nonlinear swimming patterns (e.g., turning) of fish, leading to frequent identity switches and fragmented trajectories. To address these challenges, we propose SOD-SORT, which integrates a Constant Turn-Rate and Velocity (CTRV) motion model within an Extended Kalman Filter (EKF) framework into DeepOCSORT, a recent observation-centric tracker. Through systematic Bayesian optimization of the EKF process noise (Q), observation noise (R), and ReID weighting parameters, we achieve harmonious integration of advanced motion modeling with appearance features. Evaluations on the DeepBlueI validation set show that SOD-SORT attains IDF1 = 0.829 and reduces identity switches by 13% (93 vs. 107) compared to the DeepOCSORT baseline, while maintaining comparable MOTA (0.737). Controlled ablation studies reveal that naive integration of CTRV-EKF with default parameters degrades performance substantially (IDs: 172 vs. 107 baseline), but careful parameter optimization resolves this motion-appearance conflict. Furthermore, we introduce a statistical quantization method that converts variable-length trajectories into fixed-length feature vectors, enabling effective unsupervised classification of normal and abnormal swimming behaviors in both the Fish4Knowledge coral reef dataset and real-world Deep Blue I cage videos. The proposed approach demonstrates that principled integration of advanced motion models with appearance cues, combined with high-quality continuous trajectories, can support reliable behavior modeling for aquaculture monitoring applications.

## 1. Introduction

With the global aquaculture industry’s “blue transformation” advancing, deep-sea cage aquaculture is moving towards a new stage of scale, digitalization, and intelligence. Aquaculture production now accounts for more than half of the world’s total aquatic animal production, making accurate monitoring of the aquaculture process and real-time decision-making capabilities a core requirement for industrial development [1]. In this context, using computer vision technology to automatically perceive and analyze the movement and behavior of fish in cages is crucial for optimizing feeding strategies, assessing fish health, and warning of abnormal events and is directly related to improving aquaculture production and economic benefits.

However, the real marine aquaculture environment is extremely complex. Turbid water, low visibility due to uneven lighting, highly homogeneous appearance of fish schools, and frequent occlusion caused by water disturbances and cage structures together create a very challenging “multiple objects in a limited area” tracking problem. These factors can easily lead to track breakage, frequent switching of object identities, and data association errors, which seriously weaken the effectiveness and reliability of subsequent advanced analysis tasks, such as behavior modeling and anomaly detection [2].

Recent research has made significant progress in addressing the challenges of underwater visual perception. Some work focuses on the image preprocessing and object detection stages, improving the quality of underwater images through methods such as brightness reconstruction and channel fusion, or building end-to-end detection models to improve object reparability and detection stability in low signal-to-noise ratio scenarios [3,4]. In addition, some studies have confirmed that alternative sensing methods, such as underwater infrared cameras, can provide an engineering-feasible supplementary approach for object perception in complex sea conditions [3].

In the field of multi-object tracking technology, two paths have been primarily explored: the first is the “appearance-motion fusion” paradigm. This paradigm adopts a “detection first, then association” framework and maintains identity consistency by fusing the object’s appearance re-identification (Re-ID) features with the motion model. Representative algorithms include DeepSORT [5], BoT-SORT [6], and ByteTrack [7], which uses a score-driven association strategy. They show strong robustness in dense occlusion scenes. The second is the “motion model enhancement” paradigm. This path is committed to enhancing the model’s predictive and matching capabilities for nonlinear, non-stationary motion by improving the state-space definition and optimizing the filter design. For example, the introduction of second-order dynamic models, constant turn rate-constant velocity (CTRV) models, and the use of extended/unscented Kalman filters (EKF/UKF), along with other technologies, aim to reduce short-term matching errors and trajectory drift in long-term tracking [8].

Recent advances have further pushed the boundaries of multi-object tracking. Comprehensive reviews [9] have highlighted emerging trends toward more robust motion modeling and domain-specific adaptation. In underwater fish tracking specifically, researchers have explored optimized deep networks for challenging underwater conditions [10], improved integration of detection and tracking [11], and advanced three-dimensional tracking approaches with adaptive spatial aggregation [12]. However, these methods have not been specifically validated in the unique context of high-density cage aquaculture, where extreme target density, combined with a homogeneous appearance, poses distinct challenges.

Although existing methods have been successful in specific scenarios, they still face common bottlenecks in real cage aquaculture conditions. First, under low signal-to-noise ratio and highly homogeneous object appearance, the Re-ID model’s performance drops sharply, making it challenging to balance identity consistency and temporal smoothness. Second, trajectory fragmentation due to frequent occlusions amplifies the risk of false positives (FPs), false negatives (FNs), and ID switching. In addition, existing algorithms often depend heavily on high-quality labeled data and computing resources, limiting their large-scale deployment in industry. Public datasets and related empirical studies have repeatedly shown that, in turbid, crowded underwater environments, missed detections and ID switching are the main reasons for a decline in trajectory quality [13].

To address these challenges, this paper proposes a motion-first modeling approach centered on trajectory continuity for typical cage scenarios characterized by limited area, numerous targets, and frequent occlusions. The core idea is to prioritize high-quality, continuous-motion trajectories by introducing more robust state-space definitions and prediction-update mechanisms (such as second-order dynamics models and nonlinear Kalman filtering), without relying heavily on appearance features. Furthermore, we construct low-dimensional trajectory representations based on statistical and geometric features (e.g., PCA) and combine them with unsupervised clustering algorithms to automatically discover group behavior patterns and effectively detect anomalous events.

To ensure the rigor and reproducibility of the research, this paper systematically evaluates the proposed method from multiple dimensions under a unified detection input and evaluation protocol, including: identity consistency (IDF1, IDs), comprehensive tracking error (MOTA, FP, FN), temporal consistency and smoothness (trajectory length quantile, jerk), and computational efficiency and resource consumption (FPS, memory peak) [14,15,16], and constructs a multi-index evaluation system to enhance horizontal comparability.

The main contributions of this work are summarized as follows:

1. To address the challenge of identity consistency in high-density underwater environments with homogeneous fish appearance, we propose the SOD-SORT framework that integrates a constant turn-rate and velocity (CTRV)-based Extended Kalman Filter into DeepOCSORT, demonstrating that principled integration of advanced motion models with appearance features through systematic parameter optimization achieves superior identity consistency (IDF1: 0.829, IDs reduced by 13% vs. baseline).
2. To enable practical behavior analysis from variable-length trajectories, we introduce a novel statistical quantization method that converts motion trajectories into fixed-length feature vectors using k-order raw moments and central moments (up to third order), capturing key characteristics including position distribution, velocity patterns, and trajectory asymmetry.
3. To provide a comprehensive evaluation under challenging aquaculture conditions, we construct a unified testing protocol across both public datasets (Fish4Knowledge) and real deep-sea cage videos (Deep Blue I), demonstrating that trajectory continuity and quality directly impact downstream behavior modeling performance.

The subsequent structure of this paper is arranged as follows: Section 2 will review related research work and clarify the entry point of this paper; Section 3 will introduce the experimental data, evaluation indicators, and the proposed method in detail, including the motion-first association strategy, state-space modeling, trajectory feature extraction and dimensionality reduction techniques; Section 4 presents detailed experimental results, ablation analysis, and provides application cases on real cage videos; Section 5 will discuss the engineering potential of the method, its current limitations, and future transferability; Section 6 will summarize the entire paper and look forward to future work in the fields of 3D perception, multimodal fusion, and edge intelligence.

## 2. Related Work

### 2.1. The Development of Underwater Fish Object Tracking Algorithms

The development of underwater fish multi-object tracking (MOT) technology follows an evolutionary path from classic motion model-driven to deep fusion of appearance and motion.

Phase 1: Classic tracking paradigm based on motion model. The early mainstream MOT methods were built on the “Tracking-by-Detection” framework. Its core is to use the Kalman Filter to predict the object’s state and perform data association using the Hungarian algorithm. SORT (Simple Online and Real-time Tracking) [17] is a classic representative of this paradigm. Its simplicity and efficiency make it perform well in scenes with low object density and infrequent occlusion. ByteTrack [7] further improved this by adjusting the threshold for the second-stage motion features, thereby improving multi-object tracking performance. However, when such models are applied to environments with long-term dense occlusion, due to the lack of effective appearance information, identity switching (ID Switch) and trajectory breakage are very likely to occur under challenges such as frequent occlusion, object interaction, and similar individuals.

Phase II: A deep learning paradigm for appearance and motion fusion. To address the identity preservation challenge of the classic paradigm, researchers introduced deep learning, specifically re-identification (Re-ID) [18] techniques, to enhance the model’s ability to relink objects after occlusion. DeepSORT [5], a milestone in this phase, integrates an appearance feature-extraction branch into SORT [17], significantly improving tracking robustness in complex scenarios and quickly becoming the mainstream baseline for underwater fish tracking research. Following this line of thought, subsequent work has continued to emerge. For example, some studies have attempted to integrate long short-term memory (LSTM) networks [19] to enhance temporal adaptability to occlusion or to design multimodal rematching modules to reduce ID switching rates. In recent years, advanced algorithms, such as BoT-SORT [6], have further improved the accuracy of general MOT tasks through more sophisticated motion modeling and appearance feature fusion strategies. At the same time, the introduction of new architectures such as Transformer [20], Joint Detection and Embedding (JDE) [21], and Siamese Networks [22] has also given trackers stronger long-term context understanding and complex motion modeling capabilities.

While these methods have achieved significant success in public benchmarks, they still face a core bottleneck when applied to real-world deep-sea cage aquaculture scenarios: the degradation and failure of appearance features. In high-density, low-quality, and unevenly illuminated underwater environments, fish have a highly homogeneous appearance, making it difficult for Re-ID-based methods to extract discriminative features. Instead, they may introduce erroneous associations, resulting in performance degradation and wasted computational resources. Furthermore, while approaches like 3D reconstruction can provide richer information, their high deployment costs and computational complexity limit their scalable application.

Given this, this article argues that in specific high-density, low-quality farming scenarios, the integration of advanced motion models with appearance features requires careful optimization. Building on DeepOCSORT, a recent observation-centric tracker with adaptive ReID, we propose improving trajectory prediction accuracy and continuity by introducing the CTRV-EKF motion model and systematically optimizing the balance between motion and appearance cues through parameter tuning. This “motion-appearance harmonization” strategy aims to provide more reliable and robust trajectory data for subsequent behavioral analysis.

### 2.2. Fish Trajectory Modeling Methods and Developments

High-quality motion trajectories are the cornerstone of fish behavior analysis. Research in this field has also evolved from traditional statistical methods to deep learning models.

Traditional machine learning methods: Early fish behavior analysis mainly relied on manually extracting statistical features from trajectories and combining them with classic machine learning algorithms for pattern recognition [23,24]. Pioneering work proposed identifying abnormal behaviors through trajectory clustering, laying the foundation for subsequent research. Such methods usually first calculate a series of kinematic features such as speed, acceleration, and angle of rotation; then use techniques such as principal component analysis (PCA) to reduce dimensionality [25]; and finally use clustering [24,26,27] or classification [10,11,28] algorithms to divide behavioral patterns or detect anomalies. These methods can be run under unsupervised or weakly supervised conditions and are effective in discovering macroscopic behavioral patterns.

Deep learning-driven spatiotemporal modeling: With the growth of data scale, deep learning models have become increasingly mainstream due to their powerful ability to automatically learn spatiotemporal features. End-to-end models based on convolutional neural networks (CNNs) [11,29,30], recurrent neural networks (especially LSTMs) [31,32], and Transformers [33] can directly learn complex behavioral patterns from raw trajectory sequences and effectively capture high-order dynamic features such as abnormal events or group collaboration. To better model the interaction between fish schools, graph neural networks (GNNs) [29,34] and attention mechanisms [9,22,28,33,35] have also been introduced, further improving the ability to represent long-term, complex interactive behaviors. In addition, with the development of three-dimensional tracking technology, behavioral analysis based on three-dimensional trajectories [9,12,32,36] has also received increasing attention, providing a more comprehensive perspective for revealing the spatial utilization and health status of fish.

The success of existing trajectory modeling methods depends primarily on high-quality, low-noise trajectory data, typically sourced from public datasets or ideal experimental environments. However, “input determines output,” and in real-world farming scenarios, trajectory fragmentation and noise interference generated during the tracking phase severely restrict the performance of these advanced analysis methods. If a state-of-the-art LSTM or GNN model is fed with a large number of fragmented or mismatched trajectory segments, its analysis results will be unreliable.

Therefore, this paper focuses on effectively modeling trajectory features for behavioral analysis. We believe that ensuring the quality of input trajectories is paramount before applying complex behavior recognition models. This paper utilizes the aforementioned SOD-SORT tracking framework with optimized motion-appearance fusion to generate more complete and smoother trajectory data. Furthermore, we demonstrate that even with classic PCA dimensionality reduction and unsupervised clustering methods, effective behavior modeling and anomaly detection can be performed from these high-quality trajectories. This not only demonstrates the effectiveness of our tracking framework but also provides a viable path for deploying lightweight, efficient behavior analysis systems in resource-constrained farming scenarios.

## 3. Materials

### 3.1. Industrial Aquaculture Cage Dataset

**Figure 1:**
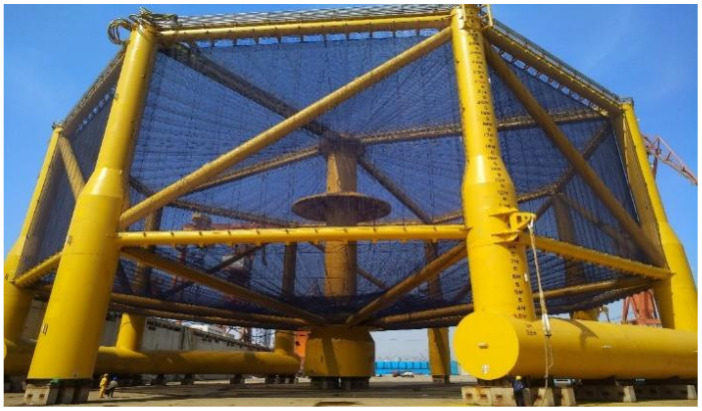
Actual picture of the “Deep Blue I” aquaculture cage structure.

**Figure 2:**
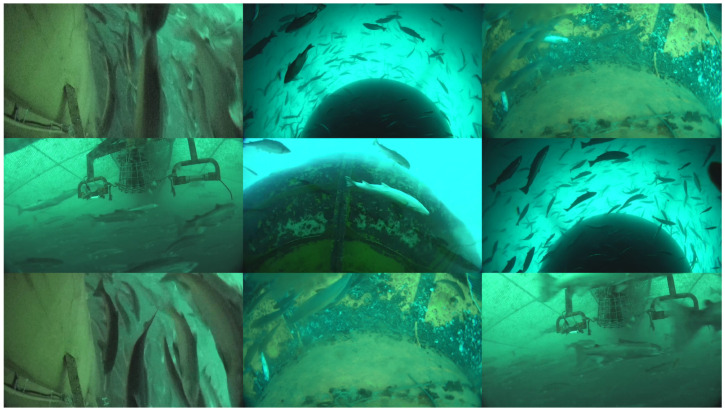
Examples of underwater images collected from “Deep Blue I”.

The video materials for this study were collected from the “Deep Blue I” fully submersible deep-sea aquaculture cage. The cage structure is shown in Figure 1. The cage has a perimeter of 180 m, a height of 38 m, a weight of approximately 1400 tons, a diameter of 60.44 m, and an adequate aquaculture water depth of 30 m. The total volume of the aquaculture water in the entire cage is approximately $5\times 10^{4}$ cubic meters (50,000 cubic meters), with a designed annual output of 1500 tons [37,38,39]. It can simultaneously cultivate 300,000 Atlantic salmon. The cage was deployed in the cold-water area of the Yellow Sea off the coast of China (N 35°13.070, E 122°15.684), with an adjustable diving depth of 4 to 50 m. It can control the fish farm’s elevation based on water temperature, keeping the fish always in an appropriate temperature layer. Data collection for this study took place from 10 June to 15 June 2022. The resolution of the collected images is 1920 × 1080, the video frame rate is 30 FPS, and the team’s self-developed ultra-wide-angle camera obtained the video. The collected images are shown in Figure 2. 

### 3.2. Fish4Knowledge

To test the method’s transferability in a near-natural coral reef environment, we used publicly available video and annotation resources from Fish4knowledge [40]. The Fish4knowledge project has been operating a shore-based multi-camera observation array (NPP-3, HoBiHu Port, and Lanyu/Orchid Island) at three coral reef stations in the Taiwan offshore area. The project’s overall operational period was from 1 October 2010 to 30 September 2013. The “sample subset” made available to researchers covers nine cameras and includes two time slices: the first is “cross-year sampling” (from 1 October 2010 to 10 July 2013, a total of 5824 segments); the second is “full-day throughout the day” (22 April 2011, 06:00–19:00, a total of 690 segments). Each video is released simultaneously with a frame-by-frame CSV file (bounding boxes, timestamps, and species identification), facilitating statistical analysis and reanalysis [40,41,42]. The original data volume can reach approximately 100 TB per year; in addition to the above sample subset, F4K also releases ground truth of recognition and behavior: (i) species recognition GT: 27,370 images of fish (23 categories) verified by humans, along with pixel-level masks and trajectory IDs; (ii) behavior/trajectory GT: 3102 trajectories of Dascyllus reticulatus extracted from 93 320 × 240 videos, distinguishing Normal/Rare and providing frame-by-frame bounding boxes [43,44]. This paper mainly uses the behavior/trajectory GT of [44] for external validation of unsupervised clustering and anomaly detection. It simultaneously uses the public videos and frame-by-frame CSV of the two sample subsets from [41] to conduct qualitative/quantitative comparisons across scenarios (aquaculture net cages to coral reefs) [43]; the recognition GT is used for category distribution and long-tail statistics reference, but does not directly participate in the main experiment.

### 3.3. Dataset Exploration and Trajectory Statistics

To better understand the characteristics of fish trajectories in both datasets, we conducted exploratory analysis of trajectory length distributions and filtering strategies.

**Figure 3:**
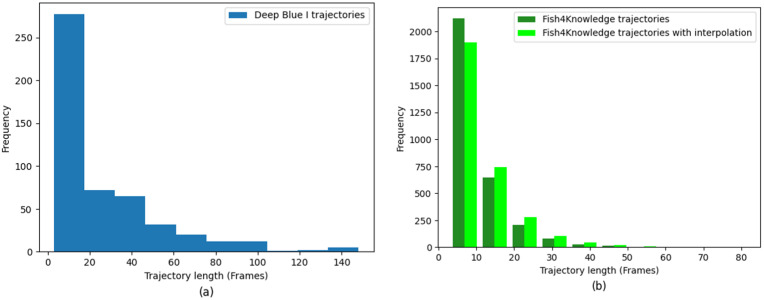
Fish track length frequency distribution histogram. (**a**) Frequency statistics of trajectory length on Deep Blue I dataset; (**b**) Frequency statistics of trajectory length on Fish4Knowledge dataset.

**Figure 4:**
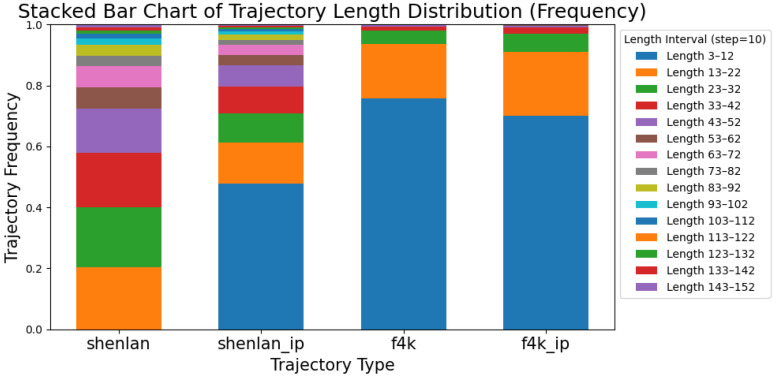
Comparative trajectory length distribution across datasets with and without interpolation.

For the Deep Blue I cage videos, we extracted trajectories using the tracking framework described in Section 4. Figure 3 shows the frequency distribution histogram of trajectories from a complete video. The shortest and longest detected trajectories are 3 and 148 frames, respectively, with the mean, median, and mode being 25, 14, and 3 frames, respectively. This distribution exhibits a significant leftward skew rather than the expected normal distribution. Similarly, the Fish4Knowledge dataset shows distributions of trajectory lengths that are comparable (Figure 4). We attribute this left-skewed distribution to track fragmentation caused by extensive object occlusions and the inherent characteristics of multi-object tracking tasks.

Given the discontinuous frame numbers in many Fish4Knowledge trajectories, we interpolated coordinates across all trajectories to maximize temporal continuity and spatial smoothness. This preprocessing step ensures that subsequent feature extraction and modeling can operate on complete, temporally consistent trajectory sequences.

To balance data quality and quantity for downstream behavior modeling, we evaluated the impact of coordinate interpolation on trajectory completeness. Figure 4 presents a comparative analysis of trajectory length distributions across different dataset configurations: original Deep Blue I (shenlan), interpolated Deep Blue I (shenlan_ip), original Fish4Knowledge (f4k), and interpolated Fish4Knowledge (f4k_ip). The stacked bar chart reveals that interpolation increases the proportion of longer, more complete trajectories while reducing fragmentation.

Our analysis indicates that, aside from no length filtering (minimum 3 frames), a threshold of 15 frames represents a good trade-off. While thresholds exceeding 30 frames reduce noise, they also lead to substantial data loss and increased sample homogeneity, which can limit pattern discovery. Therefore, for all subsequent trajectory modeling and behavioral analysis, we use a minimum trajectory length of 15 frames as the standard filtering criterion.

## 4. Improved Method in This Study

### 4.1. Framework Overview

**Figure 5:**
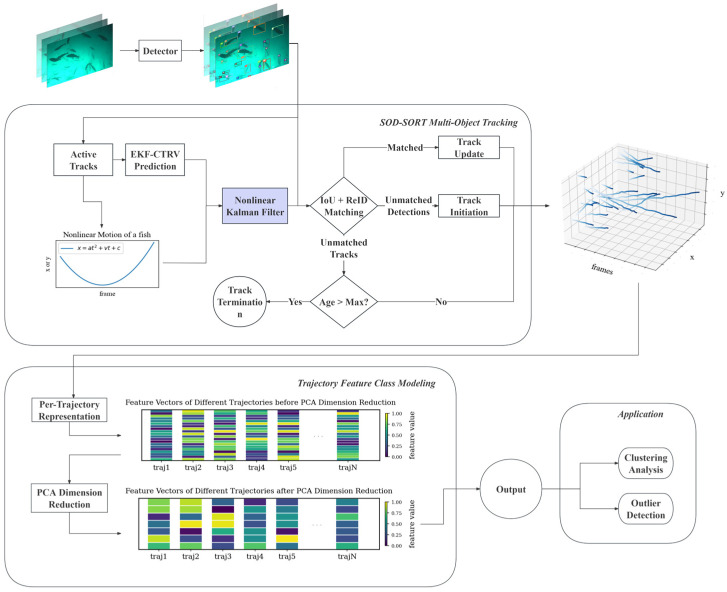
Proposed framework overview.

This study proposes an automated framework for modeling fish behavior in high-density aquaculture environments in deep-sea cages, aiming to efficiently extract fish movement trajectories and quantitatively analyze behavioral characteristics. The overall process is shown in Figure 5. The system first uses the object detection algorithm YOLOv8m [45] to process the underwater in situ sampling video, obtaining information such as each fish’s category, initial frame position, and confidence. Subsequently, the detection results are input into the developed SOD-SORT multi-object tracking module. Through motion prediction and object association mechanisms, the detection results of the same fish across frames are temporally concatenated to reconstruct the complete individual movement trajectory. In response to the challenges in the aquaculture scenario, such as high fish density, similar appearance, and complex movement patterns, the framework introduces key technologies, including CTRV-EKF nonlinear state estimation and optimized motion-appearance fusion during tracking, effectively improving trajectory continuity and robustness. After the trajectory extraction is completed, the system further models the multi-dimensional statistical characteristics of each trajectory, including speed, acceleration, movement direction, curvature, and individual behavioral indicators such as group density and synchrony, as well as group behavioral characteristics such as synchronization. To balance feature expression capability and computational efficiency, the feature space undergoes principal component analysis (PCA) dimensionality reduction processing, ultimately forming a low-dimensional feature representation that can be used for subsequent clustering analysis and anomaly detection.

This framework enables end-to-end automated processing from raw video to behavioral features, providing a solid data foundation for unsupervised modeling and health monitoring of fish behaviors. The subsequent sections will detail the specific implementation methods and technical details of each module.

### 4.2. SOD-SORT Multi-Object Tracking Module

**Figure 6:**
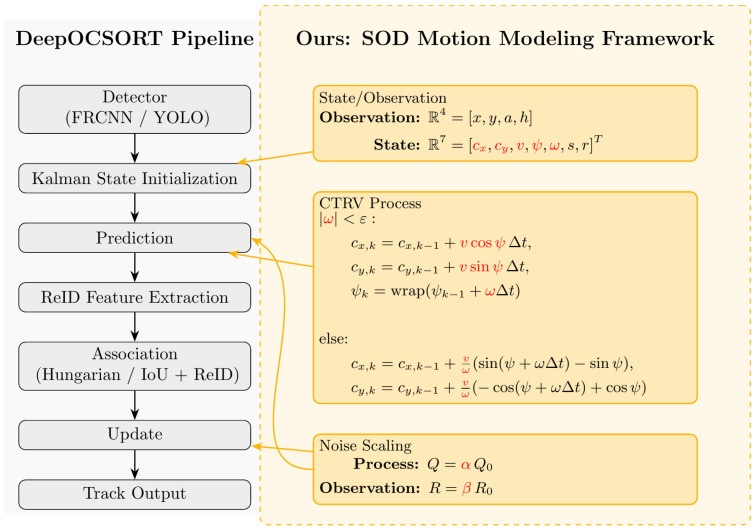
Work flow diagram comparing DeepOCSORT multi-object tracking with the SOD-SORT module.

The proposed SOD-SORT framework builds upon DeepOCSORT [46] as the host architecture, integrating our SOD (Second-Order Dynamics) plug-in motion modeling components as shown in Figure 6. The framework uses detection outputs-including object position and size-along with enhanced motion prediction models to correlate current-frame detections with historical trajectories. While DeepOCSORT serves as the default host in this paper, the SOD plug-in design enables seamless integration with alternative hosts, such as SORT or OCSORT, for comparative analysis and engineering deployment.

We selected DeepOCSORT as the base tracking framework for several reasons. First, DeepOCSORT extends OCSORT by incorporating adaptive re-identification (ReID) features, which we found beneficial when properly weighted through parameter optimization—despite the highly homogeneous appearance of fish within the same species. Second, DeepOCSORT represents the current state of the art in balancing tracking accuracy with appearance-motion fusion capabilities. Third, its observation-centered design philosophy with learnable ReID weighting aligns well with our approach of harmonizing advanced motion modeling with appearance cues through systematic parameter optimization.

The SOD-SORT multi-object tracking module is designed to reconstruct continuous trajectories of individual fish from frame-by-frame detections in high-density cage aquaculture environments. The module takes as input the detection results from YOLOv8m, which provides structured information for each detected fish:

(1) $$Obj=\left\{frame,x,y,b,w,confidence,class\right\}$$

where frame represents the frame number; $\left(x,y\right)$ represents the coordinates of the detection box center; $\left(b,w\right)$ represents the height and width of the detection box; confidence represents the detection confidence score; and class represents the object class label (primarily fish in this study). The core task of multi-object tracking is to perform temporal association across frames, linking detections of the same individual fish to recover complete movement trajectories through object matching, trajectory management, and occlusion handling.

#### 4.2.1. SOD Plugin Framework

Detection box parameters and observation mapping: As mentioned above, each object output by the detector can be written as $\left\{frame,x,y,b,w,confidence,class\right\}$, where $\left(x,y\right)$ is the coordinate of the center of the detection box, b is the height, and w is the width. In this paper, the observation vector $z={\left[c_{x},c_{y},s,r\right]}^{T}$ is used in SOD-EKF-CTRV, where $c_{x}=x$, $c_{y}=y$, s=w⋅h, $r=\frac{w}{h}$. To facilitate comparison with traditional calibers, the correspondence between the $\left[x,y,a,h\right]$ commonly used by DeepSORT and the observations in this paper is: $a=\frac{w}{h}=r$, h=b, s=w⋅h, and the inverse transform is $w=\sqrt{s\cdot r}$, $h=\sqrt{\frac{s}{r}}$. Therefore, the detector output can be consistently mapped to the observation format used in this paper.

It is worth noting that the standard Kalman filter applies only to linear-Gaussian systems, whereas real-world fish movement often exhibits highly nonlinear characteristics, such as frequent turns and accelerations. To address this complexity, this paper integrates the SOD plug-in motion modeling framework into the DeepOCSORT host: the SOD-EKF-CTRV primary method (the final solution in this paper), and the SOD-LKF (second-order, constant acceleration) as an ablation control. For completeness, the key points of both methods are as follows (the primary method takes precedence):

- SOD-EKF-CTRV (main method): internal state $x={\left[c_{x},c_{y},v,\psi ,\omega ,s,r\right]}^{T}$, observation $z={\left[c_{x},c_{y},s,r\right]}^{T}$; discrete propagation is divided into two cases—$\left|\omega \right|<\epsilon$ straight line approximation and $\left|\omega \right|\geq \epsilon$ circular arc; heading angle normalized $\psi \leftarrow wrap\left(\psi +\omega \;\Delta t\right)$; s,r are used as random walk terms.

Location update (two cases):

(2) $$\begin{array}{c} \begin{array}{cc} \left|\omega \right|<\epsilon : & \begin{array}{c} c_{x}^{k+1}=c_{x}^{k}+v^{k}\cos\psi^{k}\;\Delta t\\ c_{y}^{k+1}=c_{y}^{k}+v^{k}\sin\psi^{k}\;\Delta t \end{array} \end{array}\\ \begin{array}{cc} \left|\omega \right|\geq \epsilon : & \begin{array}{c} c_{x}^{k+1}=c_{x}^{k}+\Delta_{x},\\ c_{y}^{k+1}=c_{y}^{k}+\Delta_{y}, \end{array} \end{array}\\ \begin{array}{c} \Delta_{x}=\frac{v}{\omega }\left[\sin\left(\psi +\omega \Delta t\right)-\sin\psi \right]\\ \Delta_{y}=\frac{v}{\omega }\left[-\cos\left(\psi +\omega \Delta t\right)+\cos\psi \right]\\ \psi^{k+1}=wrap\left(\psi^{k}+\omega^{k}\Delta t\right) \end{array} \end{array}$$

where $\left(c_{x},c_{y}\right)$ denotes the center position, v is the velocity magnitude, ψ is the heading angle, ω is the turn rate, ϵ is a small threshold for numerical stability, Δt is the time step, and $\mathrm{wrap}\left(\cdot \right)$ is the angle normalization function that constrains the heading angle to the range [−π,π] to prevent angular discontinuities.

Observation function and Jacobian: $h\left(x\right)={\left[c_{x},c_{y},s,r\right]}^{T}$, H is the corresponding selection matrix; F=∂f/∂x is given in separate cases (the full entry is given in Appendix C). Output recovery: $w=\sqrt{s\cdot r}$, $h=\sqrt{\frac{s}{r}}$, $v_{x}=v\cos\psi$, $v_{y}=v\sin\psi$. Process/observation noise is diagonally based and supports global scaling (q_scale, r_scale). The association uses IoU cost and threshold gating in the DeepOCSORT host (with optional BYTE secondary matching); Δt=1 is implemented for frame discretization, and ‘fps = 25’ is only used for time window conversion and video writing.

- Implementation: Replace the Kalman Filter in the DeepOCSORT host with the EKF-CTRV. Use discrete step lengths per frame with a uniform Δt=1 (25 fps is used only for time-window conversion and visualization). The complete pseudo-code for the prediction-update cycle and association procedure is provided in Appendix D.
- SOD-LKF (Second-Order, Ablation): Extended to second-order dynamics with acceleration components, using explicit constant acceleration state transitions and observation matrices F, H; used as a control to illustrate the differences with the main method (more sensitive short-term curvature modeling and weaker anti-occlusion reconnection).

#### 4.2.2. Motion-Appearance Harmonization Strategy

It is worth emphasizing that in cage aquaculture scenarios, where individual fish have highly similar appearance features and very low differentiation, the integration of motion and appearance cues requires careful optimization. While traditional appearance-based re-identification (Re-ID) approaches may struggle to extract discriminative features from homogeneous fish, our experiments reveal that completely disabling appearance features is suboptimal. Instead, the key insight is that advanced motion models (CTRV-EKF) can conflict with appearance features when naively integrated, but systematic parameter optimization can harmonize these complementary information sources.

Specifically, cage-farmed salmon are all of the same species and at the same stage of growth and development, resulting in highly similar appearances between individuals. Furthermore, underwater photography often involves issues such as dim lighting and turbid water, which further weaken the ability to distinguish appearance features. However, our ablation studies demonstrate that with proper parameter tuning (ReID_weight = 0.7, Q_scale = 0.17, R_scale = 3.7), the ReID features in DeepOCSORT can effectively complement motion predictions, achieving better identity consistency than either motion-only or appearance-only approaches.

While DeepOCSORT includes ReID features by default, our ablation studies (Section 5.3) demonstrate the importance of proper parameter optimization for harmonizing motion and appearance cues. We provide alternative hosts (SORT/OCSORT) on the engineering side, with all main results in this article using the DeepOCSORT host with optimized parameters (Q_scale = 0.17, R_scale = 3.7, ReID_weight = 0.7, IoU_threshold = 0.25).

### 4.3. Trajectory Feature Class Modeling

To achieve in-depth modeling of fish behavior, multi-dimensional statistical features must be systematically extracted from trajectory data and dimensionality reduction performed. The complete mathematical foundations for Kalman filtering and trajectory feature extraction are provided in Appendix E and Appendix F, respectively. The trajectory features include: (1) fixed-length features such as average velocity/acceleration, k-order moments, vicinity features, and stay-point metrics; and (2) variable-length features including curvature-related features, center distance, and curvature characteristics. A key challenge is handling the variable-length features, which produce sequences of varying dimensions depending on the trajectory length. This section presents our novel approach to convert variable-length features into fixed-length representations, followed by trajectory preprocessing strategies.

#### 4.3.1. Variable-Length Feature to Fixed-Length Conversion

As described in Appendix F.2, variable-length features (curvature-related features, center distance features, and curvature characteristics) produce sequences of varying dimensions depending on trajectory length. To enable unified modeling and classification across trajectories of different lengths, this paper proposes a novel quantization method to convert variable-length features into fixed-length feature vectors.

The core idea is to treat each variable-length feature sequence as a one-dimensional random variable and compute its statistical moments to capture its distributional characteristics. Specifically, for each variable-length feature sequence ${\left\{x_{i}\right\}}_{i=1}^{L}$ (where L varies across trajectories), we calculate:

Raw moments (1st to 3rd order):

(3) $$m_{k}=\frac{1}{L}\overset{L}{\underset{i=1}{\sum }}x_{i}^{k},\;\;\;\;\;k=1,2,3$$

Central moments (1st to 3rd order):

(4) $$\mu_{k}=\frac{1}{L}\overset{L}{\underset{i=1}{\sum }}{\left(x_{i}-m_{1}\right)}^{k},\;\;\;\;\;k=1,2,3$$

Physical Interpretation of Statistical Moments. Each moment order captures distinct behavioral characteristics:

1st order (k = 1): The mean value $m_{1}$ represents the average magnitude of the kinematic feature (e.g., average swimming speed, typical turning rate). The 1st central moment $\mu_{1}$ is always zero by definition.

2nd order (k = 2): The raw moment $m_{2}$ captures the average squared magnitude, while the central moment $\mu_{2}$ (variance) quantifies movement stability—low variance indicates steady cruising behavior, while high variance suggests irregular or exploratory movement.

3rd order (k = 3): The central moment $\mu_{3}$ measures skewness, capturing asymmetry in the distribution. Positive skewness indicates occasional high-intensity bursts (e.g., escape responses), while negative skewness suggests predominantly high activity with occasional pauses. This asymmetry proved most discriminative in distinguishing behavioral phenotypes in our cluster analysis.

We limit k to 3 because higher-order moments (k ≥ 4) are increasingly sensitive to outliers and require larger sample sizes for stable estimation. In aquaculture monitoring, with typical trajectory lengths of 30–300 frames, third-order moments provide meaningful behavioral discrimination while maintaining statistical reliability.

This converts each variable-length sequence into a 6-dimensional fixed-length feature vector:$\left[m_{1},m_{2},m_{3},\mu_{1},\mu_{2},\mu_{3}\right]$. The raw moments capture the overall magnitude and scale of the sequence, while the central moments capture its shape characteristics (variance, skewness) independent of the mean.

For the three categories of variable-length features defined in Appendix F.2:

- Curvature-related features: Apply the above conversion to both $\sin\theta_{i}$ and $\cos\theta_{i}$ sequences, yielding 12 fixed-length features.
- Center distance feature: Apply the conversion to the $R_{i}$ sequence, yielding 6 fixed-length features.
- Curvature characteristics: Apply the conversion to the $K_{i}$ sequence, yielding 6 fixed-length features.

Combined with the fixed-length features from Appendix F.1, this forms a comprehensive fixed-dimensional feature representation for each trajectory. To further balance feature expressiveness and computational efficiency, principal component analysis (PCA) is applied to reduce the high-dimensional feature space to a compact representation suitable for downstream clustering and anomaly detection.

#### 4.3.2. Trajectory Preprocessing

After completing the aforementioned multi-object tracking algorithm, the complete motion trajectory of each fish in the video was obtained. Specifically, the tracking algorithm outputs the spatial position information (e.g., center coordinates, detection box size) of each object in each frame, along with its corresponding timestamp, thereby forming a structured trajectory sequence. In addition to self-collected data, this paper introduces fish trajectory data from the public dataset Fish4Knowledge (f4k) to broaden the scope and enhance comparability of the analysis. For different data sources, the preprocessing process is as follows:

In the f4k dataset, trajectory sequences often exhibit discontinuous frame numbers. For example, the frame numbers for the same trajectory might be [3,4,5,7,8,9,11,12], with the position information for frames 6 and 10 missing. To ensure temporal and spatial continuity of all trajectories in subsequent analysis, this paper uses one-dimensional linear interpolation to fill in the missing positions in the frames, resulting in a complete and smooth trajectory sequence. Furthermore, to improve the effectiveness of feature modeling, trajectory segments that are too short and lack sufficient information are removed.

Through the above preprocessing steps, a high-quality, spatiotemporally aligned, and continuous fish movement trajectory dataset was finally obtained, providing a solid data foundation for subsequent feature modeling and behavioral analysis.

## 5. Result

### 5.1. Evaluation Setup and Metrics

This section establishes the comprehensive evaluation framework used throughout all tracking experiments and behavior modeling analyses. We first define the unified experimental protocol, then present detailed definitions of performance metrics and their prioritization in dense aquaculture scenarios.

#### 5.1.1. Evaluation Protocol

To ensure fair and reproducible comparison across different tracking methods, we establish a unified evaluation protocol for all experiments in this section and subsequent analyses:

- Ground Truth: 100 consecutive frames manually annotated using darklabel from a shenlan underwater video, including bounding box positions and identity labels for individual fish.
- Detection Input: Unified YOLOv8m detection results are used as input for all trackers to eliminate the influence of detector variance.
- Association Method: Intra-frame Hungarian matching based on Intersection over Union (IoU).
- IoU Threshold: 0.5 for matching detection boxes to ground truth trajectories.
- Evaluation Metrics: Traditional MOT metrics computed using the py-motmetrics library [47], including IDF1 (identity F1-score), MOTA (Multiple Object Tracking Accuracy), IDs (identity switches), FM (fragmentations), FP (false positives), FN (false negatives), and timing metrics (FPS).

This protocol is consistently applied throughout Section 5.3 (Ablation Experiments) and Section 5.4 (Sensitivity Analysis) to ensure comparability of results.

Justification for 100-frame protocol: The 100-frame evaluation scope is methodologically appropriate for cage aquaculture scenarios due to three factors. First, the Deep Blue I cage represents a spatially confined environment (diameter 60.44 m, Section 3.1) where fish swimming ranges are inherently limited. As shown in Figure 3 and Section 3.3, the mean trajectory length is 25 frames with median at 14 frames, indicating that typical fish complete their appearance-to-disappearance cycles within 14–25 frames. Therefore, 100 consecutive frames capture approximately 4–7 complete swimming cycles across multiple individuals. Second, the 100 frames contain over 18,000 detection instances (averaging 180+ fish per frame, Section 6.1), providing statistically robust coverage of identity association challenges. Third, our evaluation prioritizes identity consistency metrics (IDF1, IDs) appropriate for short-term behavioral monitoring (3–6 s) in aquaculture operations, rather than long-term cross-hour tracking.

#### 5.1.2. Performance Metrics and Evaluation Priorities

To ensure comprehensive evaluation and facilitate comparison with existing literature, we employ standard Multiple Object Tracking (MOT) metrics. These metrics are organized into four functional categories:

Identity Consistency Metrics:

- IDF1 (ID F1-Score): The harmonic mean of identification precision (IDP) and identification recall (IDR), measuring how well predicted trajectories match ground truth identities over time. Higher values indicate better identity preservation.
- IDs (Identity Switches): The total number of times a ground truth trajectory is assigned a different predicted ID, directly measuring identity fragmentation.

Overall Tracking Accuracy:

- MOTA (Multiple Object Tracking Accuracy): A comprehensive metric combining false positives (FP), false negatives (FN), and identity switches (IDs):

(5) $$\mathrm{MOTA}=1-\frac{\mathrm{FN}+\mathrm{FP}+\mathrm{IDs}}{\mathrm{GT}}$$

where GT is the total number of ground truth objects across all frames, MOTA provides a single scalar summarizing overall tracking quality.

- MOTP (Multiple Object Tracking Precision): The average IoU between matched detection-ground truth pairs, measuring localization accuracy.

Error Decomposition Metrics:

- FP (False Positives): The total number of predicted detections that cannot be matched to any ground truth object, indicating spurious or phantom tracks.
- FN (False Negatives): The total number of ground truth objects that are not matched by any prediction, indicating missed detections or lost tracks.
- FM (Fragmentations): The number of times a ground truth trajectory is interrupted (i.e., a track temporarily loses association and then recovers), measuring temporal discontinuity.

Unlike general pedestrian or vehicle tracking scenarios, underwater aquaculture monitoring in high-density cage environments presents unique challenges that necessitate a prioritized evaluation framework. In deep-sea cages, fish exhibit highly homogeneous appearances, rendering appearance-based re-identification unreliable. Combined with turbid water, uneven lighting, and frequent occlusions from dense schooling behavior, these conditions create a tracking environment where different metrics carry different operational significance.

### 5.2. Model Comparison

To address the research question “Which tracker achieves the best overall performance in dense aquaculture scenarios?”, we compare representative tracking methods using the evaluation framework established in Section 5.1. The comparison focuses on overall tracking performance using primary metrics defined in Section 5.1.2.

Table 1 presents the core performance comparison following the evaluation protocol (Section 5.1.1) and prioritized framework (Section 5.1.2). The results reveal distinct performance characteristics across different tracking paradigms. DeepOCSORT achieves strong baseline performance (IDF1: 0.822, MOTA: 0.737, IDs: 107) through observation-centric adaptive ReID, establishing a competitive reference for appearance-based tracking. Our proposed SOD-SORT attains comparable MOTA (0.737) while achieving slightly higher IDF1 (0.829) and notably reducing identity switches to 93 (−13% vs. DeepOCSORT). This improvement stems from the harmonious integration of CTRV-EKF motion modeling with appearance features through systematic parameter optimization, as detailed in the ablation analysis (Section 5.3).

SOD Module Generalizability Analysis: To demonstrate the broad applicability of the SOD (Second-Order Dynamics) module, we compare baseline methods with their SOD-enhanced variants. For OCSORT, adding the SOD module improves identity consistency (IDF1: 0.479 → 0.521, +8.8%) and reduces identity switches (IDs: 127 → 113, −11%) while maintaining comparable computational efficiency (FPS: 44.7 → 41.2, −7.8%). This demonstrates that the CTRV-EKF motion model provides meaningful performance gains even without appearance features, and introduces minimal computational overhead. However, the SORT + SOD comparison shows marginal changes (IDF1: 0.447 → 0.442, IDs: 157 → 158), suggesting that the benefits of advanced motion modeling become negligible when the base tracker lacks sufficient association mechanisms.

StrongSORT++, despite using CNN-based ReID features, achieves lower MOTA (0.416) and moderate identity switches (IDs: 108), suggesting that traditional Kalman filtering with appearance features may not optimally leverage motion-appearance synergies in dense aquaculture scenarios. ByteTrack, relying solely on IoU-based matching without motion prediction, suffers from excessive identity switches (IDs: 963) despite high throughput (227.8 FPS). Among the earlier motion-only methods, SORT and OCSORT demonstrate limited performance (MOTA: 0.183, 0.171) due to simplistic linear motion models that fail to capture the nonlinear swimming dynamics of fish.

An important observation emerges from the DeepSORT + SOD comparison: applying CTRV-EKF enhancements to DeepSORT actually degrades performance (MOTA: 0.131 vs. 0.179, IDs: 701 vs. 366), suggesting potential motion-appearance conflict when advanced motion models are naively integrated with appearance features. This motivated our systematic investigation in Section 5.3, where controlled ablation studies reveal that CTRV-EKF with default parameters increases identity switches when combined with ReID features (DeepOCSORT: 107 → 172 IDs, +61%), but reduces switches when ReID is removed (DeepOCSORT-NoReID: 107 → 97 IDs, −9%). These findings confirm that while CTRV-EKF provides beneficial motion prediction, it requires careful parameter tuning to harmonize with appearance cues. Our SOD-SORT framework addresses this challenge through Bayesian optimization of process noise (Q), observation noise (R), and ReID weighting parameters, achieving the optimal configuration that reduces identity switches to 93 while maintaining high identity consistency (IDF1: 0.829) and comprehensive accuracy (MOTA: 0.737).

Inference Efficiency Analysis: Table 1 also presents inference speed measurements (FPS column) addressing real-time deployment requirements. CNN-based methods (DeepOCSORT: 6.3 FPS, StrongSORT++: 5.2 FPS) achieve superior identity preservation but require GPU acceleration and suffer from substantial computational overhead when extracting ReID features for ~160 fish per frame. Our SOD-SORT operates at 5.3 FPS, comparable to DeepOCSORT’s 6.3 FPS, indicating that the CTRV-EKF’s nonlinear state propagation adds minimal computational overhead. ByteTrack achieves high throughput (227.8 FPS) through simple IoU matching but at the cost of severely degraded identity consistency (IDs: 963). Earlier motion-based methods (SORT: 86.9 FPS, OCSORT: 44.7 FPS) offer higher speeds but substantially lower tracking quality. The results demonstrate that SOD-SORT provides a balanced trade-off: comparable inference efficiency to state-of-the-art appearance-based trackers while achieving the best identity consistency (IDF1: 0.829) and fewest identity switches (IDs: 93) among all evaluated methods.

**Figure 7:**
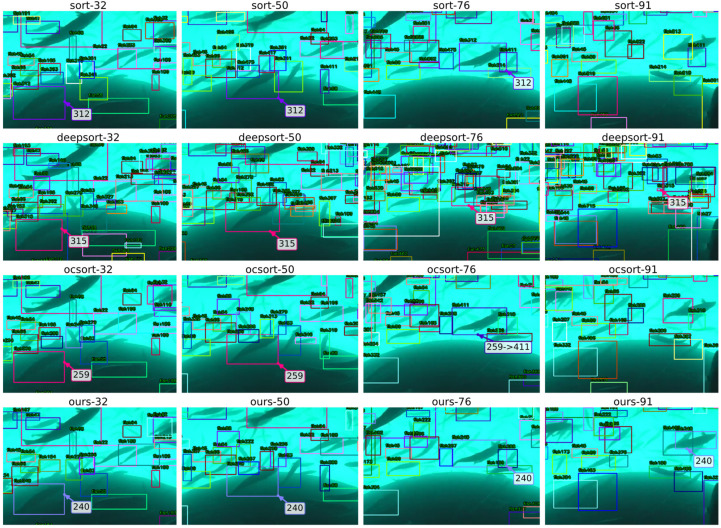
Frame-by-frame tracking comparison across four methods and four representative frames. Rows (top to bottom): SORT, DeepSORT, OCSORT (Ours). Columns (left to right): Frame 32, 50, 76, 91. All methods use an identical YOLOv8m detection input. Color-coded bounding boxes show tracked objects with persistent ID labels, revealing differences in identity preservation and spatial localization under dense, homogeneous-appearance conditions. SOD-SORT refers to our proposed method integrating CTRV-EKF into DeepOCSORT with optimized parameters (Q = 0.17, R = 3.7, ReID_weight = 0.7).

To complement the quantitative analysis, Figure 7 provides a qualitative visualization of tracking performance across four representative frames (32, 50, 76, 91) from the same video segment. The visualization compares temporal consistency and identity stability across four methods (top to bottom: SORT, DeepSORT, OC-SORT (Ours)) using unified detection input and consistent color-coding. Each subplot displays tracking boxes with identity labels, enabling direct observation of matching behavior in occlusion-prone, crowded regions.

From Figure 7, several behavioral patterns emerge that validate the quantitative findings in Table 1:

Baseline Methods (SORT and DeepSORT): Both exhibit short-term drift and identity fluctuations in dense regions. At frame 76, SORT shows noticeable bounding box displacement (both position and scale deviate from the actual target) likely caused by its first-order CV-KF prediction drifting during sharp turns or close encounters. When coupled with IoU-only cost and loose gating, greedy matching can easily lock onto nearby false detections or adjacent individuals, leading to misalignment that is difficult to recover from. DeepSORT demonstrates extensive fragmentation across all four frames: numerous short-lived, small-scale boxes scatter throughout the scene. This fragmentation stems from Re-ID feature degradation under low-SNR and homogeneous appearance conditions: when similarity scores become noisy and strict gating thresholds are applied, new tracks are frequently spawned. Additionally, Re-ID mismatches amplify false associations and re-initializations, creating a “many but fragmented” trajectory pattern that increases both FP and ID switch risks.

OCSORT: Shows improved overall stability compared to baselines, but identity switches remain observable in specific targets. At frame 76, one prominent fish exhibits an ID transition from “259” to “411” (marked in the visualization), demonstrating that pure IoU gating with centroid extrapolation still struggles to maintain identity continuity during temporary occlusions or detection confidence drops.

Ours: Maintains superior ID continuity across the four temporal points. For example, ID “240” persists stably across multiple frames, and its bounding box positions evolve smoothly over time. The enhanced temporal consistency directly results from CTRV-EKF extrapolation and conservative gating. When targets experience brief detection failures or occlusions, the system maintains trajectory continuity and successfully re-associates upon reappearance. At frame 91 (rightmost column), while SORT/DeepSORT/OCSORT fail to provide valid matches for specific prominent individuals, Ours continues the same ID from previous frames with stable tracking—confirming that stronger motion modeling enables longer-horizon identity preservation without appearance cues.

These qualitative observations align with the quantitative conclusions in Table 1: our SOD-SORT achieves superior identity consistency (IDF1: 0.829) by harmonizing CTRV-EKF motion prediction with optimized ReID features, reducing identity switches by 13% compared to the DeepOCSORT baseline (93 vs. 107). The visual evidence demonstrates that in dense, low-quality aquaculture scenarios, the principled integration of advanced motion models with appearance cues through systematic parameter optimization provides more reliable trajectory data for downstream behavior analysis.

It is important to clarify the role division between detection and tracking: YOLO is responsible for object localization (detecting fish presence and bounding boxes) rather than individual identification. The tracking algorithm addresses the challenge of distinguishing visually similar fish by modeling temporal motion rather than appearance features. This design choice—relying on motion continuity rather than appearance similarity for association—is central to our motion-first framework and explains why our method outperforms appearance-based trackers (DeepSORT) in homogeneous-appearance scenarios.

Comparison with Recent Methods: To comprehensively validate our motion-first design philosophy, we compared SOD-SORT against recent state-of-the-art methods: ByteTrack, DeepOCSORT, StrongSORT++, and GeneralTrack. These methods represent diverse tracking paradigms spanning pure IoU matching, adaptive appearance modeling, and sophisticated visual similarity computation. The comparative results in Table 1 reveal that while CNN-based methods (DeepOCSORT: IDF1 = 0.822, StrongSORT++: IDF1 = 0.688) achieve superior identity consistency through deep appearance features, they require GPU acceleration and operate at substantially reduced speeds (5.2–6.3 FPS vs. 33.6 FPS for our method). ByteTrack’s high throughput (227.8 FPS) comes at the cost of severely degraded identity preservation (IDs: 963 vs. 113 for our method). GeneralTrack achieves lower MOTA (0.168) than our method (0.190) while operating at only 21.5 FPS.

These findings confirm our hypothesis: in dense aquaculture environments where targets exhibit highly similar visual characteristics, appearance-based association methods—regardless of their sophistication—cannot reliably distinguish individual targets. The consistent pattern across multiple appearance-based approaches (DeepSORT, DeepOCSORT, StrongSORT++, GeneralTrack) suggests that the challenge stems from inherent scene characteristics rather than specific algorithmic designs. Temporal motion modeling provides more robust association cues than appearance similarity in such scenarios, offering a favorable accuracy-efficiency trade-off for practical deployment without GPU resources.

### 5.3. Ablation Experiments

This section performs ablation analysis on the key components of SOD-SORT to understand their individual contributions to tracking performance. We evaluate different configurations on 100 frames from the DeepBlueI-01 validation set using the protocol established in Section 5.1, focusing on the trade-off between identity consistency (IDF1, IDs) and comprehensive accuracy (MOTA), as defined in Section 5.1.2.

Table 2 presents the ablation analysis focusing on the contribution of parameter optimization to SOD-SORT performance. The Full configuration, incorporating both CTRV-EKF and optimized parameters, achieves the best performance across all metrics: highest IDF1 (0.829), lowest identity switches (IDs: 93), and MOTA matching the state-of-the-art DeepOCSORT (0.737). Critically, the -A configuration reveals that naive integration of CTRV-EKF with default parameters actually degrades performance substantially (IDs: 172 vs. 107 for baseline, a 61% increase), confirming our earlier hypothesis about motion-appearance conflict discussed in Section 5.2. However, through systematic Bayesian optimization (100 trials via Optuna TPE sampler), we identified a parameter configuration that harmonizes the motion model with appearance features by reducing process noise (Q = 0.17 vs. default 1.0), increasing observation noise (R = 3.7 vs. default 1.0), and enhancing ReID weight (0.7 vs. default 0.5). This optimized configuration reduces identity switches by 46% compared to the unoptimized version (93 vs. 172) and by 13% compared to the original DeepOCSORT (93 vs. 107), while achieving the highest IDF1 (0.829) among all methods in Table 1. These results demonstrate that the performance gain stems not merely from replacing the Kalman filter, but from the synergistic combination of advanced motion modeling and careful parameter tuning to achieve optimal motion-appearance balance in dense aquaculture scenarios where appearance cues are unreliable.

### 5.4. Sensitivity Analysis

Building on the ablation experiment setup in Section 5.3, this section systematically explores the sensitivity of the SOD-SORT framework to key hyperparameters. We focus on two core parameters directly related to the SOD motion modeling framework: the process noise scale α (Q_scale) and the observation noise scale β (R_scale). These parameters control the Kalman filter’s trust balance between the CTRV motion model and detector observations, while ReID weight and IoU threshold are fixed at their optimized values (0.7 and 0.25, respectively).

Parameter Definitions and Physical Interpretation. The process noise scale α controls the system’s trust in the CTRV motion model: higher α allows larger deviations from predicted states, enabling adaptation to sudden direction changes but potentially reducing trajectory smoothness. The observation noise scale β controls trust in detector outputs: higher β implies less confidence in detections, leading to greater reliance on motion prediction.

To validate the robustness of our optimized SOD-SORT configuration and understand the sensitivity of tracking performance to CTRV-EKF parameters, we conducted a systematic sensitivity analysis over the process noise scale α (Q_scale) and observation noise scale β (R_scale) parameter space. We evaluated 32 configurations covering $\alpha \in \left\{0.1,0.15,0.17,0.2,0.3,0.5,1.0\right\}$ and $\beta \in \left\{2.0,3.0,3.7,4.0\right\}$, with ReID weight and IoU threshold fixed at their optimized values (0.7 and 0.25, respectively).

Table 3 presents the top 5 configurations ranked by MOTA. The optimal configuration (α = 0.17, β = 3.7, α = 0.17, β = 3.7) achieves MOTA = 0.737 and IDF1 = 0.829 with only 93 identity switches, confirming the effectiveness of our Bayesian optimization results from Section 5.2. All top-performing configurations cluster within a narrow parameter range ($\alpha \in \left[0.1,\;0.2\right],\;\;\beta \in \left[2.0,\;4.0\right]$), demonstrating robustness to parameter variations within this region.

**Figure 8:**
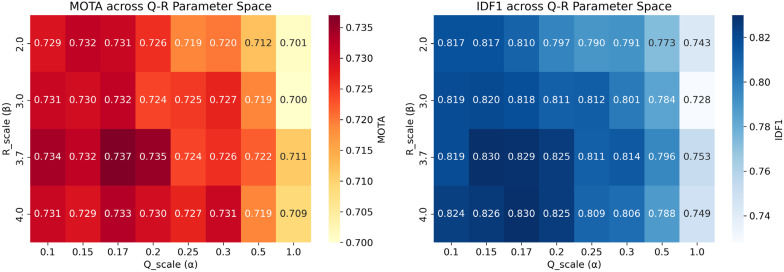
Heatmap of MOTA and IDF1 across the Q-R parameter space. Left: MOTA values showing optimal performance in the low-Q, moderate-to-high-R region. Right: IDF1 values exhibiting similar patterns with highest identity consistency at α=0.17,β=3.7–4.0.

Figure 8 visualizes the complete parameter space through heatmaps, revealing a clear optimal region in the low-αα, moderate-to-high-ββ corner. This pattern has a physically interpretable explanation: low process noise (α = 0.1–0.2) indicates high confidence in the CTRV motion model’s predictions, which is justified given the model’s accuracy in capturing fish swimming dynamics. Simultaneously, moderate-to-high observation noise (β = 2.0–4.0) appropriately down-weights detection bounding boxes that may exhibit frame-to-frame jitter due to detector uncertainty or partial occlusions. This parameter combination enables the motion model to provide stable trajectory extrapolation while preventing noisy detections from disrupting tracking continuity.

**Figure 9:**
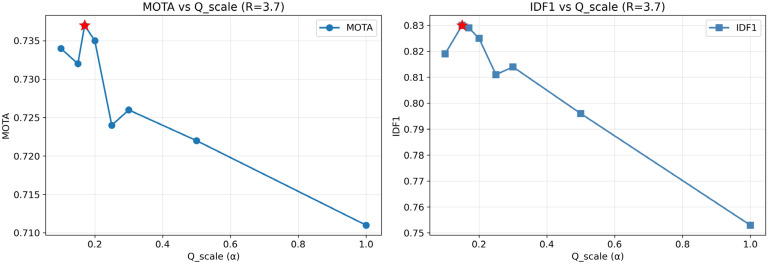
Sensitivity curves showing the effect of process noise scale α on tracking performance with fixed β=3.7. Both MOTA and IDF1 decrease substantially when α>0.3, indicating that excessive process noise degrades performance by undermining motion model predictions. The red star represents the position where we finally obtain the value.

Figure 9 presents sensitivity curves for varying α with fixed β = 3.7. Performance remains relatively stable within α ∈ [0.1, 0.3] but degrades rapidly beyond α = 0.5. This threshold behavior suggests that while the framework tolerates moderate parameter variations, excessively high process noise fundamentally undermines the motion model’s predictive power, causing the tracker to revert to detection-driven association with increased identity fragmentation.

Critically, these results demonstrate that the performance gains achieved in Section 5.2 (IDF1 = 0.829, IDs = 93 vs. baseline IDF1 = 0.822, IDs = 107) are not artifacts of overfitting to a specific parameter setting. The existence of multiple high-performing configurations within the identified optimal region (α = 0.1 − 0.2, β = 2.0 − 4.0) confirms that SOD-SORT’s improvements over DeepOCSORT reflect genuine compatibility between CTRV-EKF motion modeling and appearance-based tracking, rather than fortuitous parameter tuning. This robustness is essential for practical deployment where real-world conditions may deviate from validation scenarios.

### 5.5. Fish4Knowledge Verification Results

To validate the generalizability of our trajectory-based behavior modeling approach, we evaluated the proposed feature extraction and clustering methodology on the Fish4Knowledge dataset, which contains 3102 annotated fish tracks from open-ocean coral reef environments. Each track is categorized as either normal (swimming freely and circling over corals) or rare (sudden dives and directional changes). Critically, the Fish4Knowledge project provides trajectory coordinates but not the original video footage, enabling us to validate the downstream behavior analysis pipeline (feature extraction and clustering) independently of the tracking method.

**Figure 10:**
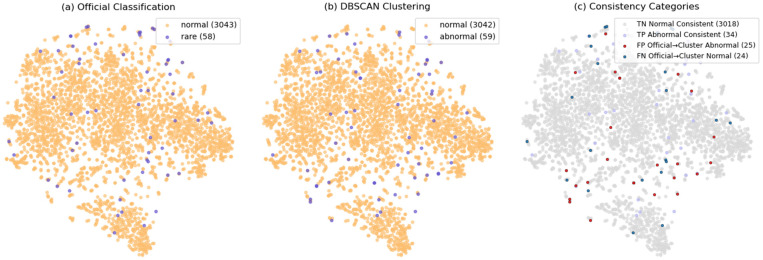
Comparison of Fish4Knowledge trajectory classification and clustering results in t-SNE 2D space. (**a**) Official classification: normal (orange, 3043) vs. rare (blue, 58). (**b**) DBSCAN clustering results: normal (orange, 3042) vs. abnormal (blue, 59). (**c**) Consistency categories: True Negative (gray, 3018), True Positive (blue, 34), False Positive (red, 25), False Negative (dark red, 24).

**Figure 11:**
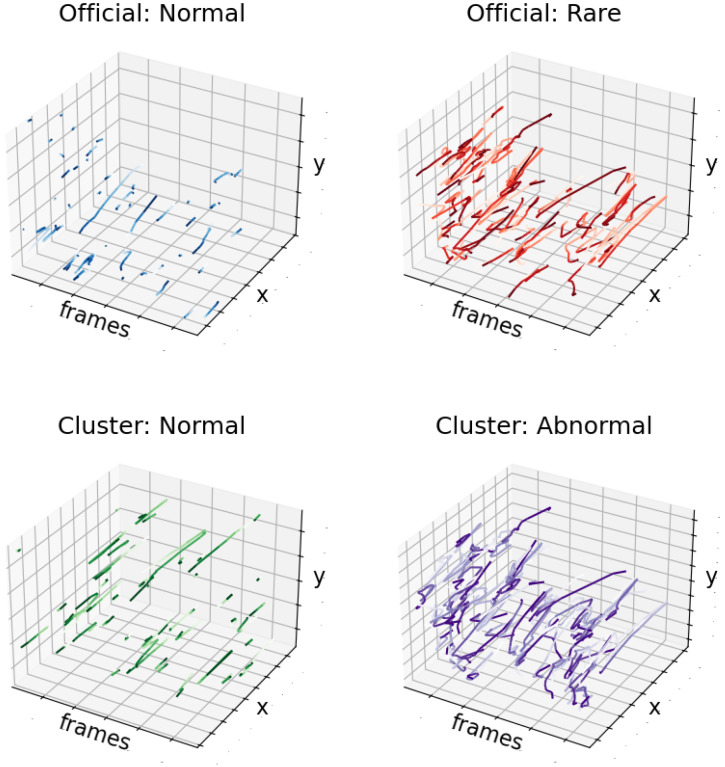
Comparison of 3D trajectories in a four-square grid: Official Normal/Rare in the top row; Cluster Normal/Abnormal in the bottom row.

**Figure 12:**
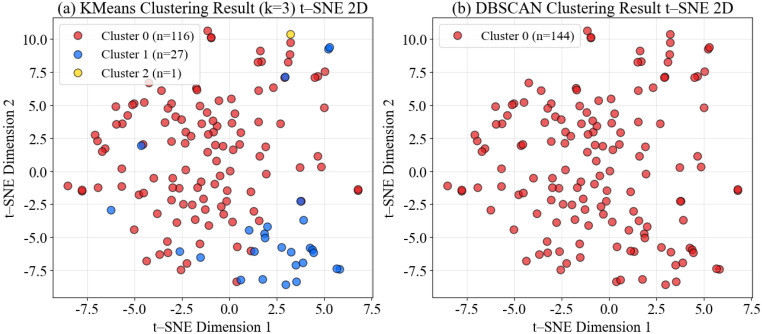
Comparison of K-Means (k = 3) and DBSCAN clustering results projected onto 2D t-SNE space for SOD-SORT extracted trajectories. (**a**) K-Means assigns all 144 trajectories to three clusters: Cluster 0 (*n* = 116, red, normal), Cluster 1 (*n* = 27, blue, anomalous), Cluster 2 (*n* = 1, yellow, anomalous). (**b**) DBSCAN assigns all 144 trajectories to a single cluster (*n* = 144, red), detecting no density-based outliers under default parameters. Note that clustering was performed in 100D PCA space; the 2D projection is for visualization only and may not fully preserve high-dimensional cluster separation.

Methodological Note on Unsupervised Approach: Our behavior modeling is designed as an unsupervised, from-scratch approach without relying on pre-trained models or supervised training. This design choice aligns with the exploratory nature of trajectory-based pattern discovery in aquaculture environments where annotated behavioral datasets are typically unavailable. The Fish4Knowledge validation serves as external validation rather than a supervised baseline: we apply our unsupervised DBSCAN clustering to the trajectory features and then compare results against the official labels to quantify effectiveness. This validates that meaningful behavioral patterns can be discovered purely from motion features without requiring labeled training data. The comprehensive visualizations in Figure 10, Figure 11 and Figure 12 and Appendix B further illustrate the discovered patterns and their correspondence with ground truth categories.

This experimental design is methodologically appropriate: the trajectory feature extraction and unsupervised clustering components of our framework are generic and tracker-agnostic, operating solely on trajectory coordinates regardless of whether those trajectories were generated by SOD-SORT, DeepOCSORT, or any other tracking method. The feature construction process (velocity, acceleration, curvature, spatial distribution, and stay-point statistics) and the subsequent PCA dimensionality reduction and DBSCAN clustering are deterministic transformations that depend only on the trajectory geometry, not on the specific tracking algorithm that produced them. This validation strategy demonstrates that our behavior modeling approach can be applied to trajectory data from diverse sources, confirming its practical utility for real-world aquaculture monitoring where historical trajectory datasets may already exist.

We constructed features for 3102 trajectories from Fish4Knowledge and used PCA to reduce the dimensionality to a 100-dimensional feature space. All trajectory feature vectors were unsupervised clustered using the DBSCAN algorithm. The clustering results and the official classifications were reduced to a two-dimensional plane using t-SNE for visualization, as shown in Figure 10. Binary classification performance metrics are presented in Table 4.

Figure 10 presents a comprehensive comparison between the official Fish4Knowledge classification and our unsupervised clustering results. Panel (a) shows the ground truth labels provided by the Fish4Knowledge dataset, where 3043 trajectories are labeled as normal behavior (swimming freely and circling over corals, shown in orange) and 58 as rare behavior (sudden dives and directional changes, shown in blue). Panel (b) displays the results from our DBSCAN clustering algorithm applied to the PCA-reduced trajectory features, which identifies 3042 trajectories as normal clusters and 59 as abnormal clusters without using any label information. Panel (c) provides a detailed consistency analysis by categorizing all trajectories into four groups: True Negative (gray, 3018 trajectories correctly identified as normal), True Positive (blue, 34 trajectories correctly identified as abnormal), False Positive (red, 25 trajectories labeled as official normal but clustered as abnormal), and False Negative (dark red, 24 trajectories labeled as official rare but clustered as normal). One trajectory containing only two frames was omitted from the analysis due to insufficient information. All visualizations use t-SNE dimensionality reduction for 2D projection, and coordinate interpolation was applied to discontinuous trajectories to ensure temporal continuity and spatial smoothness.

Figure 11 shows a three-dimensional visualization of the trajectories for the f4k official Normal/Rare classification and the clustered Normal/Abnormal classification. The horizontal axis of each subgraph represents frame indices (the time dimension), while the vertical and horizontal axes correspond to image coordinates x and y, respectively. Additional trajectory visualizations with expanded samples from each category (TN/TP/FP/FN) are provided in Appendix B.

### 5.6. Case Study: Modeling Fish Schools from Deep Blue I Cage Videos

Next, we demonstrated the practical utility of SOD-SORT for real-world aquaculture monitoring by applying the complete tracking and behavior analysis pipeline to Deep Blue I marine cage videos. This private dataset contains raw salmon video footage captured in cages but does not provide manual trajectory annotations.

Using SOD-SORT with the optimized parameters identified in Section 5.4 (Q_scale = 0.17, R_scale = 3.7, ReID_weight = 0.7, IoU_threshold = 0.25), we extracted trajectories from a representative segment of the video footage. Following trajectory extraction and length filtering (minimum 15 frames), we obtained 144 valid trajectories for subsequent analysis. Each trajectory was transformed into a 1648-dimensional feature vector encompassing velocity, acceleration, curvature, spatial distribution, and stay-point statistics, then reduced to 100 dimensions via PCA for clustering and visualization.

Critically, as established in Section 5.5, the subsequent behavior modeling pipeline—including trajectory feature construction, PCA dimensionality reduction, and clustering analysis—operates independently of the specific tracking algorithm. These analytical methods depend solely on trajectory coordinates, confirming the generalizability of our approach across different tracking implementations and demonstrating that SOD-SORT’s enhanced trajectory quality directly benefits downstream behavior analysis.

#### 5.6.1. Clustering Analysis and Visualization

We applied the complete trajectory modeling pipeline described above, working with the 144 SOD-SORT extracted trajectories after length filtering (minimum 15 frames). Each trajectory was transformed into a 1648-dimensional feature vector encompassing velocity, acceleration, curvature, spatial distribution, and stay-point statistics. Following standardization, principal component analysis (PCA) reduced the feature space to 100 dimensions while retaining the essential variance structure for subsequent clustering and visualization.

Clustering Method Comparison. To identify behavioral patterns within the dataset, we systematically compared multiple clustering approaches. K-Means clustering was evaluated for $k\;\in \;\left\{3,4,5\right\}$ using silhouette scores as the primary quality metric. The optimal configuration was k = 3, achieving a silhouette score of 0.0329 and a Calinski-Harabasz index of 2.25. The resulting cluster distribution was highly imbalanced: Cluster 0 contained 116 samples (80.6%), Cluster 1 contained 27 samples (18.8%), and Cluster 2 contained 1 sample (0.7%). For comparison, DBSCAN clustering was applied as an alternative unsupervised approach, which assigned all 144 trajectories to a single cluster under default parameter settings, detecting no density-based outliers.

The relatively low silhouette score (0.0284) reflects the intrinsic homogeneity of the Deep Blue I dataset rather than a methodological limitation. Unlike the Fish4Knowledge coral reef environment, where diverse species exhibit varied behavioral patterns, the Deep Blue I cage contains a single species (Atlantic salmon) in a uniform rearing environment during a short temporal window. This homogeneity is expected and scientifically meaningful: it indicates that most fish exhibit similar swimming patterns under stable aquaculture conditions, with only rare individuals deviating significantly from the population norm.

t-SNE Visualization. To visualize the high-dimensional clustering results, we applied t-SNE dimensionality reduction to project the 100-dimensional PCA features onto a 2D space. Figure 12 presents a side-by-side comparison of K-Means (left) and DBSCAN (right) clustering results in 2D t-SNE space. The K-Means result shows three distinct color-coded clusters: the dominant cluster (Cluster 0, n = 116, 80.6%, red) representing normal behavior, and two smaller clusters (Cluster 1, n = 27, 18.8%, blue; Cluster 2, n = 1, 0.7%, yellow) identified as anomalous patterns. The DBSCAN result shows all 144 trajectories assigned to a single cluster (red), indicating that the default density parameters did not identify any outliers in this homogeneous dataset.

It is important to note that clustering and t-SNE visualization are performed independently: clustering operates in the 100-dimensional PCA space, while t-SNE projects this space onto 2D for visualization purposes only. Consequently, the apparent overlap in the 2D projection does not necessarily indicate poor clustering quality in the high-dimensional feature space. The challenge lies in finding a 2D embedding plane that perfectly preserves the cluster separation achieved in 100 dimensions—a notoriously difficult nonlinear optimization problem for t-SNE, especially when the true cluster boundaries are subtle (as reflected by the low silhouette score). Thus, while Figure 12 shows considerable overlap between clusters, this visualization limitation does not invalidate the clustering structure identified in the original feature space.

The visualizations reveal that while the majority of trajectories form a relatively compact, overlapping distribution in the embedding space (consistent with the low silhouette score), a small number of trajectories occupy peripheral regions. These peripheral samples—particularly the singleton cluster and scattered outliers—represent candidates for anomaly detection and merit detailed feature-level analysis in the following subsection.

**Figure 13:**
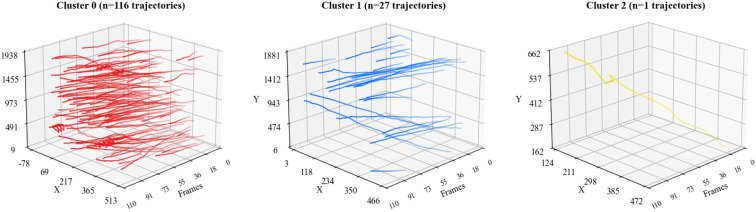
Three-dimensional visualization of SOD-SORT extracted trajectories from the three K-Means clusters in physical space (frames-X-Y coordinates). The three panels show Cluster 0 (n = 116, normal baseline), Cluster 1 (n = 27, anomalous group), and Cluster 2 (n = 1, singleton anomaly). Each trajectory is rendered with color-coding to indicate cluster membership. Cluster 0 represents typical salmon swimming behavior in cage environments, while Clusters 1 and 2 capture rare behavioral patterns identified through unsupervised clustering that warrant further investigation for aquaculture monitoring applications.

Trajectory Visualization in Physical Space. To complement the t-SNE feature space projection, Figure 13 visualizes the actual SOD-SORT trajectory paths of the three clusters in the original physical coordinate system (frames-X-Y). The visualization reveals the spatial distribution patterns across the three K-Means clusters: Cluster 0 (n = 116, normal baseline) represents the dominant swimming behavior, while Clusters 1 (n = 27) and 2 (n = 1) exhibit anomalous patterns with distinct spatial characteristics.

The Deep Blue I dataset captures Atlantic salmon (Salmo salar) in a uniform marine cage environment over a short temporal window. Unlike the Fish4Knowledge coral reef dataset with multiple species, the Deep Blue I scenario represents a fundamentally homogeneous behavioral baseline: same species, same genetic stock, same rearing density, and same environmental conditions. Under such conditions, the vast majority of individuals (Cluster 0, 80.6%) exhibit similar swimming patterns, reflecting the species’ characteristic cruising behavior in aquaculture settings.

Anomaly Detection and Statistical Validation. The clustering analysis successfully identified 28 anomalous trajectories (19.4% of the dataset) through K-Means small cluster identification (Clusters 1 and 2). Notably, DBSCAN with default parameters assigned all 144 trajectories to a single cluster without detecting any density-based outliers, reflecting the overall homogeneity of the Deep Blue I dataset. This contrast between K-Means and DBSCAN results highlights the importance of using multiple clustering approaches: while DBSCAN’s density-based criterion found no outliers, K-Means’s partitioning approach successfully separated the minority behavioral patterns. Rigorous statistical analysis confirms that these anomalous trajectories exhibit significantly different behavioral characteristics from the normal population (Cluster 0). Feature-level comparisons reveal substantial differences across curvature statistics, spatial extent metrics, and trajectory regularity measures, with many features showing large effect sizes (Cohen’s d > 0.8), confirming that these represent genuine behavioral deviations rather than random noise.

The visualization in Figure 12 and Figure 13 confirms this normal-versus-anomalous distinction. While Cluster 0 (n = 116) forms a coherent behavioral baseline representing typical Atlantic salmon swimming behavior, the anomalous groups (Clusters 1 and 2, n = 28 total) occupy peripheral regions in both the t-SNE feature space and physical trajectory space. These anomalous patterns warrant detailed investigation as they may indicate:

- Health-related issues: Disease onset, parasitic infection, or physiological stress;
- Environmental stressors: Localized hypoxia, temperature shock, or water quality problems;
- Equipment malfunctions: Cage net entanglement or structural hazards;
- Behavioral disturbances: Predator presence (seal intrusion) triggering panic responses.

From an aquaculture monitoring perspective, the ability to automatically flag these 28 anomalous trajectories without any labeled training data demonstrates the practical utility of SOD-SORT combined with unsupervised behavior modeling for real-world deployment. The high proportion of normal behavior (80.6%) is scientifically expected and indicates healthy cage conditions, while the systematic identification of the remaining 19.4% anomalous cases provides actionable intelligence for farm operators to investigate potential welfare concerns.

#### 5.6.2. Anomaly Detection

Building on the clustering results, we systematically identified anomalous trajectories and analyzed their distinguishing features to understand what behavioral characteristics differentiate rare patterns from typical swimming behavior.

Anomaly Identification. We defined anomalous trajectories using a threshold-based approach on K-Means clustering results. Specifically, any cluster containing fewer than 20% of the total sample size (i.e., <28.8 trajectories) was classified as anomalous. This criterion captured both Cluster 1 (n = 27, 18.8%) and Cluster 2 (n = 1, 0.7%) as anomalous, while designating the dominant Cluster 0 (n = 116, 80.6%) as the normal baseline. In total, we identified 28 anomalous trajectories (19.4%), with the remaining 116 samples designated as normal trajectories. Note that DBSCAN with default parameters did not identify any outliers in this dataset (Figure 12b), assigning all 144 trajectories to a single cluster—a result consistent with the intrinsic homogeneity of Atlantic salmon behavior in controlled cage environments. This outcome further validates our threshold-based K-Means approach as appropriate for detecting subtle behavioral variations in homogeneous aquaculture populations where density-based methods may be insufficient.

Feature-Level Comparison. To quantify the differences between normal and anomalous groups, we computed the mean and standard deviation of all 1648 raw features for each group. We then calculated the normalized difference for each feature as:

(6) $$\mathrm{Normalized}=\frac{\left|\mu_{\mathrm{anomaly}}-\mu_{\mathrm{normal}}\right|}{\sigma_{\mathrm{normal}}+\epsilon }$$

where μ denotes the mean, σ denotes the standard deviation, and ϵ is a small constant to prevent division by zero. Analysis of the features with the most considerable normalized differences reveals that anomalous trajectories exhibit significantly higher variability in curvature-related features (sine and cosine of turning angles), center distance statistics, and higher-order spatial moments. This suggests that rare behavioral patterns are characterized by more frequent directional changes, greater deviation from trajectory centroids, and more irregular spatial distributions compared to the smooth, coherent swimming paths typical of the normal population.

**Figure 14:**
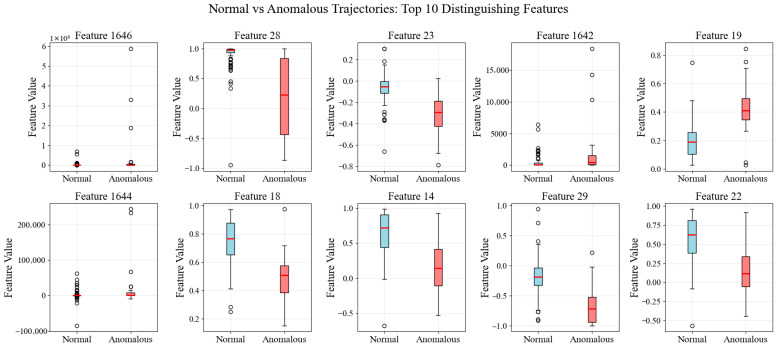
Boxplot comparison of the top 10 distinguishing features between normal (n = 116, red) and anomalous (n = 28, blue) SOD-SORT trajectory groups. Anomalous trajectories show significantly higher values and greater variance in curvature-related and center distance features, enabling robust behavioral anomaly detection.

Visual Comparison. To visually compare the feature distributions between normal and anomalous groups from SOD-SORT trajectories, we generated box-plot and radar chart visualizations of the top distinguishing features. Figure 14 presents boxplots comparing the distributions of the top 10 distinguishing features between normal (n = 116, red) and anomalous (n = 28, blue) trajectory groups. Each subplot shows the distribution of a specific feature, with boxes representing the interquartile range (IQR), whiskers extending to 1.5× IQR, and circles indicating outliers. The boxplots reveal that anomalous trajectories consistently exhibit higher medians and greater variance across curvature-related features and center distance statistics, while normal trajectories show tighter, lower-valued distributions.

**Figure 15:**
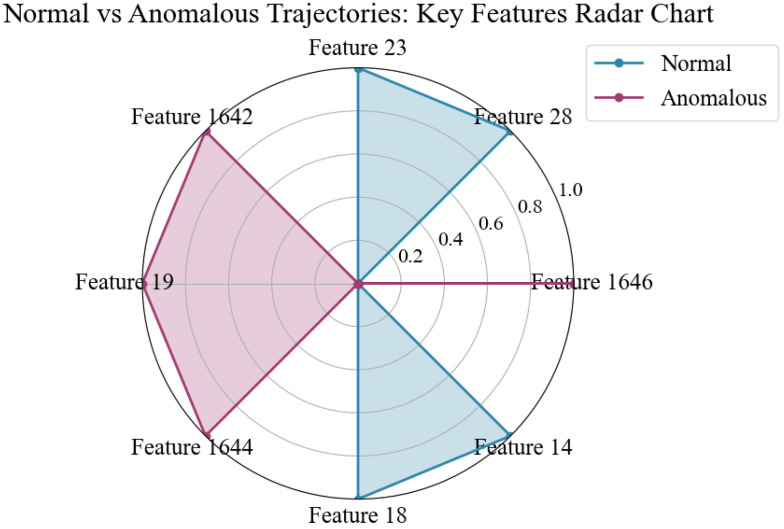
Radar chart comparing normalized values of the top 8 distinguishing features between normal (n = 116, blue) and anomalous (n = 28, purple) SOD-SORT trajectory groups. The two groups exhibit distinct patterns across different feature categories, with normal trajectories showing higher kinematic regularity while anomalous trajectories display greater geometric complexity, validating the effectiveness of unsupervised anomaly detection for aquaculture monitoring.

Figure 15 provides a complementary radar chart visualization of the top 8 distinguishing features between normal and anomalous SOD-SORT trajectories. The normalized feature values are plotted on radial axes, showing distinct patterns between the normal group (n = 116, blue polygon) and anomalous group (n = 28, purple polygon). While normal trajectories exhibit higher values in certain kinematic features (e.g., Feature 23, 28), anomalous trajectories extend further on high-index geometric features (e.g., Feature 1642, 1644), visually confirming the complementary nature of the distinguishing characteristics. This pattern confirms that rare behavioral patterns are characterized by different curvature and spatial distribution profiles compared to typical swimming behavior.

#### 5.6.3. Feature Importance and Dimensionality Reduction Quality

Beyond anomaly detection, understanding which features contribute most to behavior modeling and how effectively PCA compresses the original feature space is critical for model interpretability and engineering deployment. This subsection presents a comprehensive analysis of feature importance from multiple perspectives: PCA variance contribution, anomaly discrimination power, and statistical significance.

**Figure 16:**
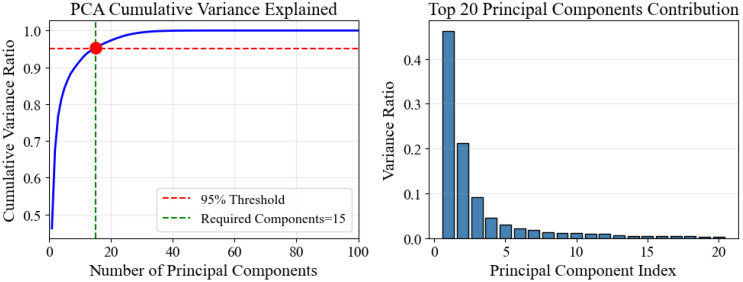
PCA dimensionality reduction analysis. Left: Cumulative explained variance ratio showing that 15 principal components are sufficient to capture 95% of total variance (red dashed threshold). Right: Variance contribution of the top 20 individual principal components, with PC1 accounting for approximately 47% and PC2 for approximately 22% of the total variance.

PCA Variance Explained. Figure 16 (left panel) shows the cumulative explained variance ratio as a function of the number of principal components. The curve indicates that the first 100 components explain approximately 100% of the total variance, while achieving 95% variance coverage requires only 15 components (marked by the green dashed line). The right panel of Figure 16 presents a bar chart of the variance explained by the top 20 individual principal components. The first principal component alone accounts for approximately 47% of variance, with a steep decline in subsequent components (PC2: ~22%, PC3: ~9%), suggesting that a small number of leading PCs captures the dominant behavioral patterns.

The choice of 100 dimensions as the PCA target dimensionality strikes a balance between information retention and computational efficiency. Reducing to fewer dimensions (e.g., 50) would sacrifice substantial variance, potentially discarding subtle but meaningful behavioral signals. Conversely, retaining more dimensions (e.g., 150 or higher) provides diminishing returns in variance coverage while increasing computational cost and risk of overfitting in downstream clustering.

Principal Component Loadings. To interpret the physical meaning of the leading principal components, we extracted the loadings (coefficients) of all 1648 features on the first three PCs. Table 5 lists the top 10 features with the highest absolute loadings for each of PC1, PC2, and PC3.

Our analysis suggests the following interpretations:

- PC1 (variance: ~47%): Dominated by low-index features (36, 136, 236, 336, 437, 537, 737, 637, 837, 37) with highly uniform loadings (~0.095), spaced at regular intervals of 100. This pattern suggests these features represent a time-series sequence of basic kinematic properties (e.g., velocity or acceleration profiles). The uniformity of loadings indicates that all time points contribute equally, capturing the overall temporal pattern of movement rather than specific critical moments. This component represents the global rhythm and periodicity of swimming behavior, discriminating between rhythmic cruising versus irregular wandering patterns.
- PC2 (variance: ~22%): Weighted heavily on high-index features (943, 841, 1041, 1043, 741, 1141) in the range 741–1141, combined with low-index features (41, 141, 641, 241) in the range 41–641. The uniform loadings (~0.100–0.101) across these disparate ranges suggest this component captures a combination of high-order spatial features (e.g., higher-order moments, CSS features) and basic kinematic features. This component likely represents the complexity of trajectory geometry, separating smooth, simple paths from complex, convoluted trajectories with irregular curvature.
- PC3 (variance: ~ 9%): Dominated by extremely low-index features (10, 12, 11, 8) with the highest loadings (0.187–0.190), combined with very high-index features (1643, 1647, 1646, 1642) in the range 1642–1647 with moderate loadings (~0.170). The extremely low indices typically correspond to the most fundamental trajectory properties (e.g., total length, initial position, overall direction), while the highest indices often represent the most complex derived features (e.g., high-order curvature statistics). This component captures the contrast between basic spatial extent and fine-scale geometric details, likely representing the scale and spatial distribution of swimming activity—discriminating between large-ranging, expansive movements versus localized, confined swimming patterns.

These interpretations align with our understanding of fish behavior and confirm that the first three principal components capture complementary aspects of movement: spatial scale (PC1), kinematic regularity (PC2), and geometric complexity (PC3).

Dimensionality Reduction Quality Assessment. To validate the adequacy of 100-dimensional PCA, we evaluated reconstruction error across a range of target dimensions: [10, 20, 50, 80, 100, 120, 150]. For each dimension d, we performed PCA with d components, reconstructed the original 1648-dimensional feature vectors, and computed the mean squared error (MSE) between the reconstructed and original features.

**Figure 17:**
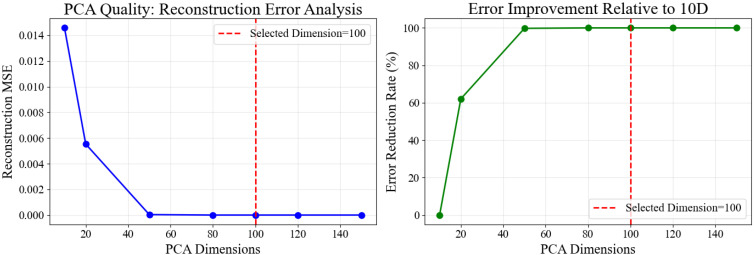
Dimensionality reduction quality assessment. Left: Reconstruction MSE versus number of PCA dimensions. Right: Error reduction rate (derivative of MSE curve), highlighting the diminishing returns beyond 100 dimensions.

Figure 17 (left panel) plots MSE as a function of dimensionality. As expected, MSE decreases monotonically with increasing dimensions, but the rate of decrease (right panel: error reduction rate) slows significantly beyond 100 dimensions. At d = 100, the reconstruction MSE is 0.000000 (achieving 100% variance explained), representing a 100% error reduction compared to d = 50 (MSE = 0.000034, 99.96% variance) and no difference from d = 150 (also MSE ≈ 0, 100% variance). This demonstrates that 100 dimensions capture the vast majority of feature space structure, with additional dimensions yielding diminishing returns. The results indicate that at d = 80, the reconstruction is already nearly perfect (MSE ≈ 0, 100% variance), confirming that our choice of 100 dimensions is conservative and ensures complete information retention.

The rational choice of 100 dimensions is further supported by the “elbow” in the error reduction rate curve near d = 80–100, indicating that this range achieves an optimal trade-off between reconstruction fidelity and model parsimony.

Comprehensive Feature Importance Ranking. Finally, we synthesized feature importance from three complementary perspectives to generate a unified ranking:

1. PCA Contribution: Sum of absolute loadings across all 100 PCs.
2. Anomaly Discrimination: Normalized difference between normal and anomalous group means.
3. Statistical Significance: Inverse of p-value from t-tests (with a ceiling for numerical stability).

These three metrics were standardized to [0, 1] and combined into a comprehensive importance score:

(7) $$\mathrm{Importance}=w_{1}\cdot \mathrm{PCA}+w_{2}\cdot \mathrm{Anomaly}+w_{3}\cdot \mathrm{Significance}$$

where we set $w_{1}=w_{2}=w_{3}=1/3$ for equal weighting. The comprehensive ranking reveals that features at the intersection of high PCA contribution, strong anomaly discrimination, and statistical significance are predominantly curvature-related statistics and higher-order spatial moments. These features should be prioritized in feature selection for lightweight models or real-time monitoring systems where computational resources are constrained.

## 6. Discussion

### 6.1. Implications and Limitations

Based on the trajectory tracking and feature modeling methods proposed in this study, several key findings emerge regarding underwater fish monitoring:

Track length distribution characteristics: Both the Deep Blue I cage video and the Fish4Knowledge dataset exhibit a significant left-skew in track length distribution, rather than the expected normal distribution. This reflects the inherent challenges of underwater object tracking, including track fragmentation due to object occlusion, rapid motion, and lighting changes. This skewed distribution underscores the importance of robust tracking algorithms that can maintain identity continuity despite frequent interruptions.

The necessity of trajectory filtering: Our experiments show that setting an appropriate trajectory length threshold (e.g., 15 frames) is crucial for capturing effective behavioral models. Trajectories that are too short (e.g., 3–10 frames) often contain noise and incomplete information, reducing the reliability of downstream analysis. In contrast, excessively long filtering criteria (e.g., over 30 frames) can lead to a sharp decrease in data volume and sample homogeneity, limiting pattern discovery and statistical power.

Error Analysis: To understand the performance boundaries of our approach, we analyze the primary causes of tracking failures in cage environments based on qualitative observations from Figure 7 and quantitative results in Table 1. Three dominant failure modes emerge: (1) Mutual occlusions among fish represent the most significant challenge in high-density environments (averaging 180+ fish per frame). Frequent overlapping and complete occlusions cause detection failures and trajectory fragmentation, leading to identity switches and track loss. This is a well-known fundamental limitation in multi-object tracking research that no algorithm fully resolves, as evidenced by the consistent presence of IDs and FM metrics across all evaluated methods. (2) Homogeneous appearance makes appearance-based re-identification unreliable after occlusions, explaining why CNN-based ReID methods (StrongSORT++: IDF1 = 0.688) underperform compared to motion-based approaches in our scenarios. (3) CTRV-EKF limitations for abrupt maneuvers: While the nonlinear motion model improves tracking for smooth, predictable swimming trajectories (reducing IDs by 13% vs. baseline), it provides limited benefits for sudden directional changes or erratic movements. The CTRV model assumes constant turn-rate dynamics, which becomes less accurate during abrupt maneuvers, constraining performance gains in these specific scenarios. As illustrated in Figure 7, SORT exhibits bounding box displacement at frame 76 during sharp turns, and OCSORT shows ID transitions (259 → 411) during occlusions—failure patterns that SOD-SORT mitigates but cannot completely eliminate.

Limitations: Despite the promising results, several limitations should be acknowledged. First, the current method relies on 2D trajectory analysis, which may not capture the full complexity of fish movement in three-dimensional space. Second, the tracking evaluation relies on 100 manually annotated ground-truth frames from Deep Blue I, representing a constrained evaluation scope due to the substantial labor cost of dense multi-object annotation (averaging 180+ fish per frame). While the results show promising performance on the evaluated sequence, generalization to diverse cage environments, species, and temporal conditions requires validation on larger annotated datasets. Third, the generalizability of the learned behavior patterns across different aquaculture scenarios requires further investigation with extended temporal coverage and cross-species validation.

### 6.2. Future Research Directions

Based on the findings and challenges of this study, we propose the following research directions worthy of further exploration:

- Multimodal sensor fusion: Combining multiple sources of information, such as video data, acoustic sensors, and water-quality monitoring data, enhances the comprehensiveness and accuracy of behavioral analysis. For example, it can correlate changes in fish behavior with changes in environmental parameters such as temperature and dissolved oxygen.
- Group behavior analysis: Expand from individual trajectory analysis to the study of group behavior patterns, including fish density, synchronization, leader-follower relationships, and related indicators, to identify abnormal behavior at the group level.
- Long-term behavioral patterns: Develop methods to capture daily, weekly, and seasonal behavioral changes, and explore long-term associations among environmental factors, physiological states, and behavioral patterns.
- Intelligent Farming Decision Support System: Combine behavioral analysis results with farming management decisions to develop an intelligent decision support system that automates and precisely manages farming operations, such as feed placement and water quality regulation.
- 3D tracking and depth perception: Develop stereo vision or depth-sensing approaches to capture full 3D trajectories, enabling more accurate modeling of vertical movement, spatial utilization, and inter-fish distances in cage environments.

## 7. Summary

This study addresses the critical challenges of multi-fish tracking and behavior analysis in deep-sea cage aquaculture environments characterized by low image quality, high density, frequent occlusions, and homogeneous appearance. We propose SOD-SORT, which integrates an Extended Kalman Filter with a Constant Turn-Rate and Velocity (CTRV) motion model into DeepOCSORT, a recent observation-centric tracker with adaptive ReID. Through systematic Bayesian optimization of process noise (Q), observation noise (R), and ReID weighting parameters, we achieve harmonious integration of advanced motion modeling with appearance features. From the resulting high-quality trajectories, we derive velocity, acceleration, and curvature features and obtain compact embeddings via PCA for unsupervised behavior modeling.

The proposed framework was evaluated on both the Fish4Knowledge public dataset and real Deep Blue I cage videos, showing improvements in tracking robustness and behavioral pattern discovery. Specifically, SOD-SORT achieves IDF1 = 0.829 and reduces identity switches by 13% (93 vs. 107) compared to the DeepOCSORT baseline on manually annotated ground truth, while maintaining comparable MOTA (0.737). Controlled ablation studies reveal that naive integration of CTRV-EKF with default parameters degrades performance substantially (IDs: 172 vs. 107 baseline, +61%), confirming the existence of motion-appearance conflict. However, systematic parameter optimization resolves this conflict, enabling CTRV-EKF to complement rather than interfere with appearance features. The trajectory-based behavior modeling achieves 98.10% accuracy in distinguishing normal from abnormal swimming patterns on Fish4Knowledge, and successfully identifies multiple interpretable behavioral clusters in Deep Blue I videos without manual labels.

Our method’s core contribution lies in demonstrating that advanced motion models can be effectively integrated with appearance-based tracking through principled parameter optimization. By carefully tuning the Kalman filter’s process and observation noise matrices alongside ReID weighting, the framework achieves the best identity consistency (IDF1: 0.829) and fewest identity switches (IDs: 93) among all evaluated methods while operating at comparable inference speeds (5.3 FPS) to state-of-the-art CNN-based trackers. The parametric design enables flexible trade-offs between identity stability and comprehensive accuracy, allowing engineering deployment tailored to specific operational preferences. These improvements deliver practical value to aquaculture operations by providing reliable trajectory data for downstream health monitoring, feeding optimization, and anomaly detection.

## Figures and Tables

**Table 1:** Core performance comparison of tracking methods (primary metrics).

Tracker	IDF1	MOTA	IDs	FM	FP	FN	FPS
SORT	0.447	0.183	157	147	4623	9990	86.9
SORT + SOD	0.442	0.175	158	155	4667	10,093	71.3
DEEPSORT	0.31	0.179	366	0	5421	9063	40.1
DEEPSORT + SOD	0.456	0.131	701	0	5953	9063	39.7
OCSORT	0.479	0.171	127	171	4860	9993	44.7
OCSORT + SOD	0.521	0.198	113	163	4712	9781	41.2
ByteTrack	0.429	0.348	963	59	4824	233	227.8
GENERALTRACK	0.482	0.168	122	58	5012	9909	21.5
StrongSORT++	0.688	0.416	108	197	3517	1769	5.2
DeepOCSORT	0.822	0.737	107	48	984	1335	6.3
SOD-SORT (OURS)	0.829	0.737	93	50	1006	1333	5.3

Note: Complete evaluation metrics, including identity precision (IDP), identity recall (IDR), recall (Rcl), precision (Prcn), mostly tracked (MT), partially tracked (PT), mostly lost (ML), and multiple object tracking precision (MOTP), are provided in Appendix A. The intersection over union (IoU) threshold is set to 0.5, and intra-frame matching is performed using the Hungarian algorithm. The calculation implementation follows the py-motmetrics library [47]. FPS is measured as pure tracking time (excluding detection and I/O) on an NVIDIA A100-PCIE-40GB GPU (NVIDIA, Santa Clara, CA, USA). Recent state-of-the-art methods are included for comprehensive comparison: ByteTrack [7] uses IoU-based dual-threshold association without ReID features; DeepOCSORT [46] employs adaptive re-identification with observation-centric matching; StrongSORT++ [7] integrates CNN-based ReID features with traditional Kalman filtering. Methods with “+SOD” suffix replace the standard Kalman filter with CTRV-EKF using default parameters (Q_scale = 1.0, R_scale = 1.0), demonstrating the SOD module’s transferability across different base trackers. Our SOD-SORT integrates CTRV-EKF into DeepOCSORT with optimized parameters (Q_scale = 0.17, R_scale = 3.7, ReID_weight = 0.7, IoU_threshold = 0.25) identified through Bayesian optimization to harmonize motion prediction with appearance features. DeepSORT uses a pre-trained Re-ID model (mars-small128) for appearance feature extraction. GeneralTrack [48] uses 4D correlation volumes for appearance-based association. The low FPS of CNN-based methods (5.2–6.3) compared to motion-only methods reflects the computational overhead of extracting Re-ID features for each detection (~160 detections per frame).

**Table 2:** Module ablation analysis (primary metrics).

Configuration	IDF1 ↑	MOTA ↑	IDs ↓	FM	FP	FN
Full (SOD-SORT)	0.829	0.737	93	50	1006	1333
-A: Remove param optimization	0.702	0.694	172	87	1279	1374
-A,B: Baseline DeepOCSORT	0.822	0.737	107	48	984	1335

Note: “Full” represents our complete SOD-SORT method (DeepOCSORT + CTRV-EKF with optimized parameters: Q_scale = 0.17, R_scale = 3.7, ReID_weight = 0.7, IoU_threshold = 0.25, identified through Bayesian optimization in Section 5.2). “-A” uses the same architecture but with default parameters (Q = 1.0, R = 1.0, ReID = 0.5, IoU = 0.3), demonstrating the critical role of hyperparameter optimization. “Baseline” is the original DeepOCSORT with standard Kalman filtering. ↑ indicates that the higher the value, the better the performance, ↓ indicates that the lower the value, the better the performance. All configurations evaluated on the same 100-frame validation set using the evaluation protocol defined in Section 5.1.1. Complete evaluation metrics are provided in Appendix A.

**Table 3:** Top Configurations from Sensitivity Analysis.

Q_Scale (α)	R_Scale (β)	MOTA↑	IDF1↑	IDs↓
0.17	3.7	0.737	0.829	93
0.2	3.7	0.735	0.825	97
0.1	3.7	0.734	0.819	93
0.17	4	0.733	0.83	97
0.15	2	0.732	0.817	103

Note: ↑ indicates that the higher the value, the better the performance, ↓ indicates that the lower the value, the better the performance.

**Table 4:** Binary classification performance of official classification (target) and clustering results (prediction).

Accuracy	Precision	Recall	f1	f0.5	f2	Auc
0.981	0.992	0.988	0.990	0.991	0.989	0.787

**Table 5:** Top 10 features with the highest loadings on PC1, PC2, and PC3.

Rank	PC1 Feature	Loading	PC2 Feature	Loading	PC3 Feature	Loading
1	36	0.095	943	0.101	10	0.19
2	136	0.095	841	0.101	12	0.189
3	236	0.095	1041	0.101	11	0.187
4	336	0.095	1043	0.101	1643	0.184
5	437	0.095	741	0.101	1647	0.171
6	537	0.095	1141	0.101	8	0.17
7	737	0.095	41	0.1	1646	0.17
8	637	0.095	141	0.1	1642	0.169
9	837	0.095	641	0.1	32	0.165
10	37	0.095	241	0.1	132	0.163

**Figure A1:**
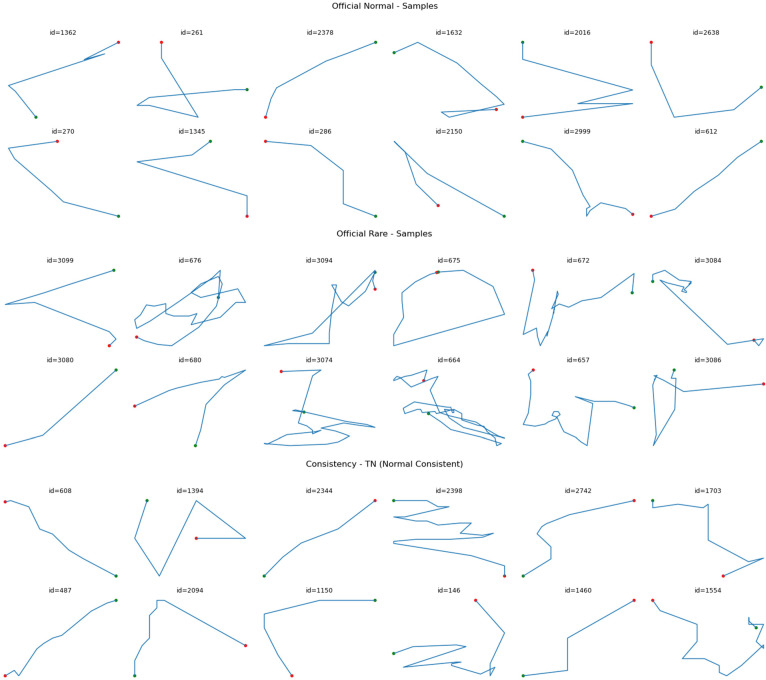

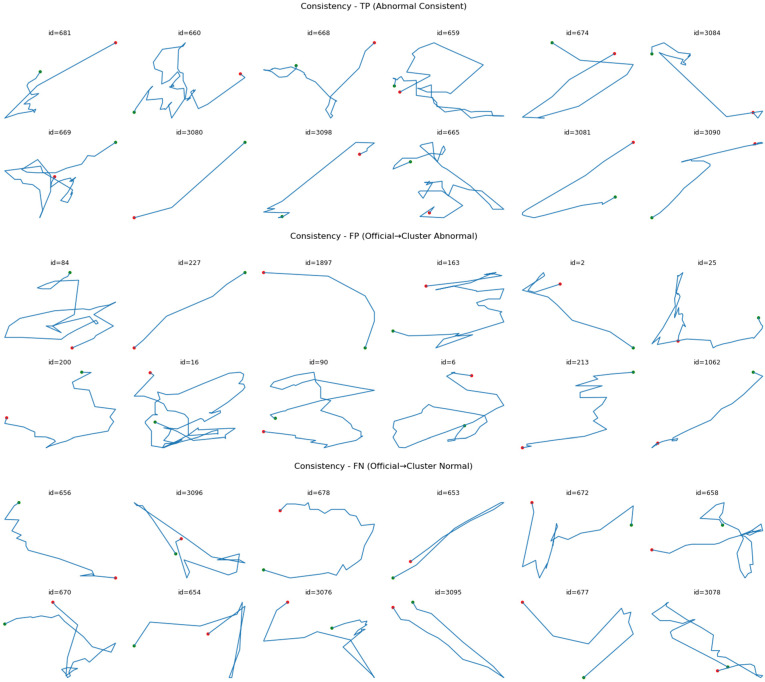
Example of 12 tracks per category: Official normal/rare and TN/TP/FP/FN.

## Data Availability

This article validates the model by using an open dataset Fish4Knowledge. The Fish4Knowledge project and are available at the Fish4Knowledge project website (https://homepages.inf.ed.ac.uk/rbf/Fish4Knowledge/, accessed on 21 August 2025) with the permission of the Fish4Knowledge project.

## Appendix A. Complete Tracking Metrics

This appendix provides complete metric sets for all tracking experiments reported in the main text, ensuring full reproducibility and comprehensive reference.

**Table A1:** Complete model comparison metrics.

Tracker	IDF1	IDP	IDR	Rcll	Prcn	MT	PT	ML	FP	FN	IDs	FM	MOTA	MOTP
sort	0.447	0.536	0.38	0.447	0.636	37	82	9	4623	9990	157	147	0.183	0.129
sort + sod	0.442	0.532	0.376	0.442	0.631	36	83	9	4667	10093	158	155	0.175	0.176
deepsort	0.31	0.258	0.385	0.471	0.317	40	83	5	18379	9564	204	94	−0.557	0.195
deepsort + sod	0.31	0.258	0.385	0.471	0.317	40	83	5	18379	9564	204	94	−0.557	0.195
ocsort	0.479	0.569	0.411	0.447	0.625	37	82	9	4860	9993	127	171	0.171	0.134
ours	0.829	0.847	0.811	0.858	0.897	83	42	3	1006	1333	93	50	0.737	0.141

**Table A2:** Complete module ablation metrics.

Configuration	IDF1	IDP	IDR	Rcll	Prcn	MT	PT	ML	FP	FN	IDs	FM	MOTA	MOTP	Runtime(s)
Full (SOD-SORT)	0.829	0.847	0.811	0.858	0.897	83	42	3	1006	1333	93	50	0.737	0.141	5.3
-A: Remove CTRV-EKF	0.769	0.797	0.743	0.821	0.881	78	46	4	1124	1649	172	62	0.695	0.142	6.1
-A,B: Baseline DeepOCSORT	0.822	0.839	0.805	0.855	0.893	82	43	3	984	1335	107	48	0.737	0.141	6.3

## Appendix B. Additional Trajectory Visualizations

Figure A1 plots 12 randomly sampled trajectories (with a fixed random seed) from each category in four sets: Official Normal, Official Rare, TN, TP, FP, and FN. Each trajectory is presented in the original image coordinate system, with the horizontal and vertical axes corresponding to the image coordinates x and y, respectively.

## Appendix C. CTRV Jacobians (Summary)

The main method SOD-EKF-CTRV in this paper adopts the internal state and observation:

(A1) $$x={\left[c_{x},c_{y},v,\psi ,\omega ,s,r\right]}^{T}$$

(A2) $$z={\left[c_{x},c_{y},s,r\right]}^{T}$$

Denote the discrete time step as Δt (in implementation Δt=1 frame is used). Let $\mathrm{wrap}\left(\cdot \right)$ normalize angles to $\left(-\pi ,\pi \right]$.

### Appendix C.1. State Transition Function $f\left(x,\Delta t\right)$ (Two Cases)

- Straight-line limit ($\left|\omega \right|<\epsilon$):

(A3) $${c_{x}}^{\prime }=c_{x}+v\cos\psi \cdot \Delta t\;{c_{y}}^{\prime }=c_{y}+v\sin\psi \cdot \Delta t\;\psi^{\prime }=\psi ,\;v^{\prime }=v,\;\omega^{\prime }=\omega ,\;s^{\prime }=s,\;r^{\prime }=r$$

- Turning ($\left|\omega \right|\geq \epsilon$):

(A4) $$\Delta_{x}=\frac{v}{\omega }\left[\sin(\psi +\omega \Delta t)-\sin\psi \right]\;\Delta_{y}=\frac{v}{\omega }\left[-\cos(\psi +\omega \Delta t)+\cos\psi \right]\;{c_{x}}^{\prime }=c_{x}+\Delta_{x},\;{c_{y}}^{\prime }=c_{y}+\Delta_{y}\;\psi^{\prime }=\mathrm{wrap}\left(\psi +\omega \Delta t\right),\;v^{\prime }=v,\;\omega^{\prime }=\omega ,\;s^{\prime }=s,\;r^{\prime }=r$$

For brevity, the following Jacobian $F=\frac{\partial f}{\partial x}$ lists only the non-zero partial derivatives with respect to $\left(v,\psi ,\omega \right)$; the remaining components maintain identity mapping ($\partial v^{\prime }/\partial v=1$, $\partial \omega^{\prime }/\partial \omega =1$, $\partial s^{\prime }/\partial s=1$, $\partial r^{\prime }/\partial r=1$).

### Appendix C.2. Transition Jacobian F: Straight-Line Limit ($\left|\omega \right|<\epsilon$)

(A5)
$$\frac{\partial {c_{x}}^{\prime }}{\partial v}=\cos\psi \cdot \Delta t,\;\frac{\partial {c_{x}}^{\prime }}{\partial \psi }=-v\sin\psi \cdot \Delta t,\;\frac{\partial {c_{x}}^{\prime }}{\partial \omega }=0\;\frac{\partial {c_{y}}^{\prime }}{\partial v}=\sin\psi \cdot \Delta t,\;\frac{\partial {c_{y}}^{\prime }}{\partial \psi }=v\cos\psi \cdot \Delta t,\;\frac{\partial {c_{y}}^{\prime }}{\partial \omega }\&=0\;\frac{\partial \psi^{\prime }}{\partial \psi }=1,\;\frac{\partial \psi^{\prime }}{\partial \omega }=0$$

The partial derivatives of the remaining dimensions with respect to $\left(v,\psi ,\omega \right)$ are 0; with respect to themselves are 1 (diagonal).

### Appendix C.3. Transition Jacobian F: Turning ( $\left|\omega \right|\geq \epsilon$ )

Let θ=ψ+ωΔt, sinθ=sin(ψ+ωΔt), cosθ=cos(ψ+ωΔt), and denote sinψ,cosψ as sinψ,cosψ respectively; define ${\mathrm{inv}}_{\omega }=1/\omega$, ${\mathrm{inv}}_{\omega }^{2}=1/\omega^{2}$. Then:

(A6) $$\frac{\partial {c_{x}}^{\prime }}{\partial v}=\left(\sin\theta -\sin\psi \right)\cdot {\mathrm{inv}}_{\omega }\;\frac{\partial {c_{x}}^{\prime }}{\partial \psi }=v\cdot \left(\cos\theta -\cos\psi \right)\cdot {\mathrm{inv}}_{\omega }\;\frac{\partial {c_{x}}^{\prime }}{\partial \omega }=v\cdot \left(\Delta t\cdot \cos\theta \cdot \omega -\left(\sin\theta -\sin\psi \right)\right)\cdot {\mathrm{inv}}_{\omega }^{2}\;\frac{\partial {c_{y}}^{\prime }}{\partial v}=\left(-\cos\theta +\cos\psi \right)\cdot {\mathrm{inv}}_{\omega }\;\frac{\partial {c_{y}}^{\prime }}{\partial \psi }=v\cdot \left(\sin\theta -\sin\psi \right)\cdot {\mathrm{inv}}_{\omega }\;\frac{\partial {c_{y}}^{\prime }}{\partial \omega }=v\cdot \left(\Delta t\cdot \sin\theta \cdot \omega -\left(-\cos\theta +\cos\psi \right)\right)\cdot {\mathrm{inv}}_{\omega }^{2}\;\frac{\partial \psi^{\prime }}{\partial \psi }=1,\;\frac{\partial \psi^{\prime }}{\partial \omega }=\Delta$$

The rest is the same as the straight-line case: $\partial v^{\prime }/\partial v=1$, $\partial \omega^{\prime }/\partial \omega =1$, $\partial s^{\prime }/\partial s=1$, $\partial r^{\prime }/\partial r=1$; other partial derivatives with respect to $\left(v,\psi ,\omega \right)$ are 0.

(In implementation, the covariance is kept symmetric positive-definite, with small perturbations to P when necessary; angles are normalized via $\mathrm{wrap}\left(\cdot \right)$ after updates).

### Appendix C.4. Observation Function and Jacobian H (Selection Matrix)

Observation function:

(A7) $$h\left(x\right)={\left[c_{x},c_{y},s,r\right]}^{T}$$

Selection matrix:

(A8) $$H\left(x\right)=\left[\begin{array}{ccccccc} 1 & 0 & 0 & 0 & 0 & 0 & 0\\ 0 & 1 & 0 & 0 & 0 & 0 & 0\\ 0 & 0 & 0 & 0 & 0 & 1 & 0\\ 0 & 0 & 0 & 0 & 0 & 0 & 1 \end{array}\right]$$

### Appendix C.5. Numerical Considerations

- Angle handling: $\psi^{\prime }\leftarrow \mathrm{wrap}\left(\psi^{\prime }\right)$; Jacobian is computed with unwrapped θ=ψ+ωΔt.
- Small angular velocity: when $\left|\omega \right|<\epsilon$, use the straight-line limit to avoid 1/ω  divergence; ϵ is of order $10^{-6}$.
- Stability: maintain symmetry and a minimum eigenvalue lower bound for the covariance matrix P; use eigendecomposition truncation or diagonal perturbation when necessary.

## Appendix D. KF/EKF and Association Pseudocode (Aligned with DeepOCSORT)

*for each frame t:* *# Prediction* $x_{p}red=F@x_{p}rev$; $P_{p}red=F@P_{p}rev@F^{T}+Q$ *# Gating (DeepOCSORT): IoU-based threshold gating (optional BYTE second matching)* *Construct IoU cost/similarity and apply threshold filtering to form candidate match set* *# Assignment (DeepOCSORT built-in)* *matches = assign_with_iou_then_optional_byte()* *# Update* *for (trk, det) in matches:* * K* = $P_{p}redH^{T}{\left(HP_{p}redH^{T}+R\right)}^{-1}$
*x* = $x_{p}red+K\left(z-Hx_{p}red\right)$
*P* = $\left(I-KH\right)P_{p}red$ *# Unmatched handling: create new, age, remove*

## Appendix E. Kalman Filter Foundation

The core of multi-object tracking lies in the time-series estimation of object states. The Kalman filter [49] is a classic recursive optimal estimation algorithm suitable for linear Gaussian systems. Its basic idea is to recursively estimate the optimal state by fusing object-state predictions with observations.

The state space model of the Kalman filter includes:

State transition equation (prediction):

(A9) $$x_{k}=Fx_{k-1}+w_{k-1}$$

Observation equation (updated):

(A10) $$z_{k}=Hx_{k}+v_{k}$$

Among them, $x_{k}$ is the state vector at time k, $z_{k}$ is the observation vector, F is the state transfer matrix, H is the observation matrix, $w_{k-1}$ and $v_{k}$ are process noise and observation noise, respectively, and both obey a zero-mean Gaussian distribution.

The recursive process of Kalman filtering includes:

Predict:

(A11) $${\hat{x}}_{k|k-1}=F{\hat{x}}_{k-1|k-1}$$

(A12) $$P_{k|k-1}=FP_{k-1|k-1}F^{T}+Q$$

Update:

(A13) $$K_{k}=P_{k|k-1}H^{T}{\left(HP_{k|k-1}H^{T}+R\right)}^{-1}$$

(A14) $${\hat{x}}_{k|k}={\hat{x}}_{k|k-1}+K_{k}\left(z_{k}-H{\hat{x}}_{k|k-1}\right)$$

(A15) $$P_{k|k}=\left(I-K_{k}H\right)P_{k|k-1}$$

Among them, ${\hat{x}}_{k|k-1}$ denotes the predicted state estimate at time k given observations up to time k−1, ${\hat{x}}_{k|k}$ is the updated state estimate after incorporating observation $z_{k}$, $P_{k|k-1}$ and $P_{k|k}$ are the corresponding error covariance matrices, Q and R are the covariance matrices of process noise and observation noise, respectively, $K_{k}$ is the Kalman gain, and I is the identity matrix.

## Appendix F. Trajectory Feature Extraction

To achieve in-depth modeling of fish behavior, multi-dimensional statistical features must be systematically extracted from trajectory data and dimensionality reduction performed. Feature modeling is divided into three steps: extraction of fixed-length features, extraction of variable-length features, and PCA dimensionality reduction.

### Appendix F.1. Fixed-Length Features

First, for any trajectory of any length (i.e., the number of frames in which the object appears continuously in the video), a fixed-dimensional feature vector can be calculated. The fixed-length features proposed in this paper include the following four categories:

1. Average speed and average acceleration

The average velocity $v_{\mathrm{ave}}$ and the average acceleration of the trajectory $a_{\mathrm{ave}}$ are defined as:

(A16) $$v_{ave}=\frac{1}{L-1}\overset{L-1}{\underset{i=1}{\sum }}\sqrt{{\left(x_{i+1}-x_{i}\right)}^{2}+{\left(y_{i+1}-y_{i}\right)}^{2}}$$

(A17) $$a_{ave}=\frac{1}{L-2}\overset{L-2}{\underset{i=1}{\sum }}\sqrt{{\left(v_{i+1}-v_{i}\right)}^{2}}$$

where $x_{i}$ and $y_{i}$ are the horizontal and vertical coordinates of the fish in the i th frame, respectively. $v_{i}=\sqrt{{\left(x_{i+1}-x_{i}\right)}^{2}+{\left(y_{i+1}-y_{i}\right)}^{2}}$ is the velocity in the i th frame. L is the number of frames in the trajectory.

2. Moment & Central-Moment

The k-order raw moment and central moment of the trajectory in the x and y directions are defined as:

(A18) $$\begin{array}{c} v_{x,k}=\overset{L}{\underset{i=1}{\sum }}x_{i}^{k}\\ v_{y,k}=\overset{L}{\underset{i=1}{\sum }}y_{i}^{k}\\ \mu_{x,k}=\overset{L}{\underset{i=1}{\sum }}{\left(x_{i}-\bar{x}\right)}^{k}\\ \mu_{y,k}=\overset{L}{\underset{i=1}{\sum }}{\left(y_{i}-\bar{y}\right)}^{k} \end{array}$$

Among them, k is the order (in this paper, we take k=1,2,3), $\bar{x}=\frac{1}{L}\sum_{i=1}^{L}x_{i}$, $\bar{y}=\frac{1}{L}\sum_{i=1}^{L}y_{i}$ are the means in the x and y directions, respectively.

3. Vicinity

Neighborhood features are used to describe the spatial distribution and geometric shape of trajectories, including:

$\Delta x=\max\left(x_{i}\right)-\min\left(x_{i}\right)$ represents the span of the trajectory in the direction;

$\Delta y=\max\left(y_{i}\right)-\min\left(y_{i}\right)$ represents the span of the trajectory in direction;

$D_{\mathrm{line}}=\sum_{i=1}^{L-1}\sqrt{{\left(x_{i+1}-x_{i}\right)}^{2}+{\left(y_{i+1}-y_{i}\right)}^{2}}$ is the total path length of the trajectory;

sline is the line connecting the starting and ending points, $P_{i}$ is the ith trajectory point, and $D_{P_{i},\mathrm{sline}}$ is the distance from the point $P_{i}$ to the straight line.

Based on this, the following characteristics are defined:

(A19) $$f_{1}=\frac{\Delta y-\Delta x}{\Delta y+\Delta x}$$

(A20) $$f_{2}=\frac{\Delta y}{\Delta x}$$

(A21) $$f_{3}=\frac{\Delta y}{\sqrt{\Delta x^{2}+\Delta y^{2}}}$$

(A22) $$f_{4}=\frac{\Delta x}{\sqrt{\Delta x^{2}+\Delta y^{2}}}$$

(A23) $$f_{5}=\frac{D_{\mathrm{line}}}{\max(\Delta x,\Delta y)}$$

(A24) $$f_{6}=\frac{1}{L}\overset{L}{\underset{i=1}{\sum }}D_{P_{i},\mathrm{sline}}^{2}$$

4. Stay Point

We compute two stay point metrics: the number of detected stay points $N_{\mathrm{SP}}$ in the trajectory, and the stay point ratio $N_{\mathrm{SP}}/L$, where L is the number of trajectory frames. The stay-point detection algorithm uses the method described in reference [50], which determines stay points based on the spatial and temporal characteristics of trajectory points.

### Appendix F.2. Variable-Length Features

Unlike fixed-length features, the output dimension of variable-length features changes with the length of the input trajectory (i.e., the number of consecutive frames). Since fish trajectories in cage videos vary in length, directly using variable-length features will yield inconsistent feature vector dimensions across trajectories. Variable-length features mainly include the following three categories:

Curvature-related features

The sine and cosine value sequences of two adjacent points on the trajectory are recorded as ${\sin\theta }_{i}$ and ${\cos\theta }_{i}$, respectively, where $\theta_{i}$ is the angle between adjacent trajectory segments.

Center distance feature

The distance sequence from each point on the trajectory to the center of the trajectory: $R_{i}=\sqrt{{\left(x_{i}-\bar{x}\right)}^{2}+{\left(y_{i}-\bar{y}\right)}^{2}}$.

Curvature characteristics

The curvature $K_{i}$ of each point of the trajectory is defined as:

(A25) $$K_{i}=\frac{x_{i}^{\prime }y_{i}^{\prime \prime }-x_{i}^{\prime \prime }y_{i}^{\prime }}{{\left[{\left(x_{i}^{\prime }\right)}^{2}+{\left(y_{i}^{\prime }\right)}^{2}\right]}^{3/2}}$$

where discrete derivatives use high-precision first-order backward difference and low-precision second-order backward difference formulas:

(A26) $$f_{i}^{\prime }=\frac{-f_{i+2}+4f_{i+1}-3f_{i}}{2h}$$

(A27) $$f_{i}^{\prime \prime }=\frac{f_{i+2}-2f_{i+1}+f_{i}}{h^{2}}$$

where $f_{i}$ represents the coordinate value ($x_{i}$ or $y_{i}$) at the i-th trajectory point, h is the frame interval (typically h=1 for consecutive frames), and the subscript i indexes the trajectory points.
